# Combined Ensemble Docking and Machine Learning in Identification of Therapeutic Agents with Potential Inhibitory Effect on Human CES1

**DOI:** 10.3390/molecules24152747

**Published:** 2019-07-29

**Authors:** Eliane Briand, Ragnar Thomsen, Kristian Linnet, Henrik Berg Rasmussen, Søren Brunak, Olivier Taboureau

**Affiliations:** 1 INSERM U1133, CNRS UMR 8251, Unit of functional and adaptive biology, Université de Paris, Paris 75013, France; 2 Department of Forensic Medicine, Faculty of Health and Medical Sciences, University of Copenhagen, 2200 Copenhagen N, Denmark; 3 Institute of Biological Psychiatry, Mental Health Centre Sct. Hans, Copenhagen University Hospital, 4000 Roskilde, Denmark; 4 Department of Science and Environment, Roskilde University, 4000 Roskilde, Denmark; 5 Novo Nordisk Foundation Center for Protein Research, Faculty of Health and Medical Sciences, University of Copenhagen, 2200 Copenhagen N, Denmark

**Keywords:** carboxylesterase 1, docking, ensemble docking, machine learning, CES1 inhibitors, adverse drug reactions, metabolism

## Abstract

The human carboxylesterase 1 (CES1), responsible for the biotransformation of many diverse therapeutic agents, may contribute to the occurrence of adverse drug reactions and therapeutic failure through drug interactions. The present study is designed to address the issue of potential drug interactions resulting from the inhibition of CES1. Based on an ensemble of 10 crystal structures complexed with different ligands and a set of 294 known CES1 ligands, we used docking (Autodock Vina) and machine learning methodologies (LDA, QDA and multilayer perceptron), considering the different energy terms from the scoring function to assess the best combination to enable the identification of CES1 inhibitors. The protocol was then applied on a library of 1114 FDA-approved drugs and eight drugs were selected for in vitro CES1 inhibition. An inhibition effect was observed for diltiazem (IC50 = 13.9 µM). Three others drugs (benztropine, iloprost and treprostinil), exhibited a weak CES1 inhibitory effects with IC50 values of 298.2 µM, 366.8 µM and 391.6 µM respectively. In conclusion, the binding site of CES1 is relatively flexible and can adapt its conformation to different types of ligands. Combining ensemble docking and machine learning approaches improves the prediction of CES1 inhibitors compared to a docking study using only one crystal structure.

## 1. Introduction

The incidence of drug failures remains high in late-stage clinical development phases, leading to a key challenge in drug discovery [1]. Essentially this reflects a lack of efficacy and clinical safety [2]. Although drug response is a complex phenotype depending on numerous genetic environmental and behavioral factors, studies have demonstrated that pharmacokinetics, and notably metabolism, can alter the response to medicine and lead to increased risk of adverse drug reactions (ADRs) through for example unanticipated drug–drug interactions (DDIs) [3]. Studies assessing the potential DDIs have been dedicated mostly to redox enzymes such as cytochromes P450, however, less has been done to rationalize hydrolytic drug metabolizing enzymes such as human carboxylesterase 1 (CES1) [4]. 

CES1 is an enzyme member of the serine hydrolase family. Among the five human carboxylesterases (CESs), CES1 is the predominant form expressed in the liver [5,6]. CES1 is implicated in the hydrolysis of many drugs (and prodrugs), transforming an ester, amide or carbamate moiety into its respective free acids, amines or alcohols [7]. It metabolizes structurally diverse compounds such as the psychostimulant methylphenidate (MPH) [8] used in the treatment of hyperkinetic disorders, commonly prescribed angiotensin-converting enzyme (ACE) inhibitors (enalapril, trandolapril, ramipril, quinapril, imidapril) [9], the anti-cancer agent (CPT-11) [10,11], the anti-influenza prodrug oseltamivir [12], and several narcotics and analgesics (cocaine, meperidine) [13,14]. CES1-mediated metabolism can yield either inactive metabolites, as in the deactivation of MPH into ritalinic acid, or active metabolites, as in the case of prodrugs activation, such as activation of oseltamivir phosphate to its active form, oseltamivir carboxylate. In addition to xenobiotic substrates, CES1 is also involved in the processing of several endogenous compounds such as fatty acids and cholesterol derivatives [15,16].

The publication of crystal structures of CES1 has greatly contributed to the understanding of the mechanism of action of the enzyme. Fourteen X-ray structures of CES1 released in the Research Collaborator for Structural Bioinformatics (RCSB) Protein Databank were all complexed with a ligand (substrate analogs, inhibitors, endogenous metabolites, and enzymatic products) [17,18,19,20]. In addition, four new X-ray structures in the *apo* state have been resolved recently [21,22]. Tremendous efforts have been devoted by examining both structural and biochemical requirements of these enzymes to hydrolyze their substrates [23,24,25], and several early studies reported different types of CESs inhibitors [26,27,28,29,30,31,32,33]. 

In-silico studies involving ligand-based approaches have been applied in order to identify therapeutic agents acting as strong CES1 inhibitors leading to potential drug–drug interactions (DDIs) [34]. Pharmacophore and QSAR methods have been applied on protease inhibitor antiviral drugs [35]. 3D-QSAR studies have been performed on a class of compounds based on benzil (1,2-diphenylethane-1,2-dione) and isatins (Indole-2,3-diones) [28,32]. Structure-based approaches such as docking and molecular dynamics simulation were also performed to elucidate the mechanisms of binding, which is essentially the key role of hydrophobic interactions in ligand binding and the flexibility of the active site to adapt to specific ligands. [29,36,37]. Finally, our previous docking studies analyzed the underlying mechanism of drug response variability resulting from CES1 polymorphism. It confirmed the critical role of the Gly143 allele in the metabolism of MPH [38,39] and suggested that the polymorphism Glu220Gly could also affect the enzyme function [40].

Overall, despite the number of clinical drugs identified as CES1 inhibitors, CES1 inhibition is still an overlooked source of DDIs. All drugs have not been systematically assessed for their inhibitory capacity on CES1. Therefore, our study is designed as an attempt to identify clinically prescribed drugs exhibiting CES1 inhibitory activity with potential for producing CES1-based drug interactions, using an approach that combines ensemble docking and machine learning methods. Previous studies have reported that ensemble docking based on molecular dynamics simulations or on multiple crystallographic structures were more successful than docking based on single conformation [41]. Furthermore, combined with a machine learning approach, it has the advantage of increasing virtual screening performance while reducing the amount of errors that would be introduced by a single method [42,43,44].

## 2. Results

### 2.1. Binding Site Description

**Figure 1:**
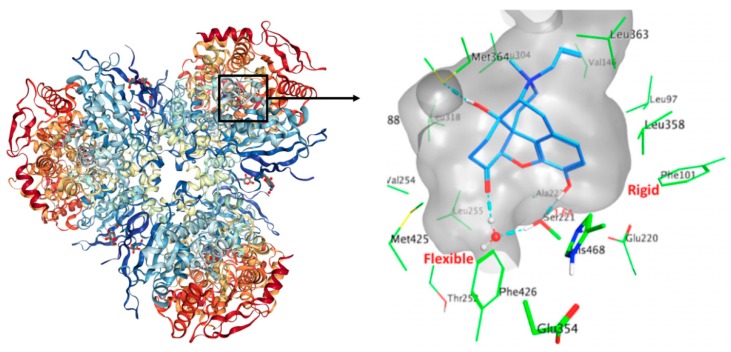
The figure on the left side represents the X-ray of CES1 complexed with naloxone (PDBID: 1mx9). The figure on the right side is a zoom on the binding site of CES1 where naloxone is located. The protein is represented as a grey transparent surface. Residue side chains within 4.5 Å of the ligand are shown as green sticks. Hydrophobic interactions are predominant. Intermolecular hydrogen bonds are shown as cyan dashes. The catalytic triad residues Ser221–Glu354–His468 located at the base of the active gorge and between the rigid and flexible pockets are shown in thick sticks. The average distance between Ser221 side chain and naloxone’s hydroxyl group is indicated with a solid red line.

CES1 exists in a trimer–hexamer equilibrium. Each monomer of the enzyme is composed of three functional domains namely a central catalytic domain, which contains the serine hydrolase catalytic triad (Ser221, His468 and Glu354), an α/β domain that stabilizes the trimeric architecture, and a regulatory domain. The active site is located at the base of a 10–15 Å deep catalytic gorge located at the interface of the three domains and is predominantly lined by hydrophobic residues. Two acidic negatively-charged residues are present in the CES1 cavity, namely, Glu220, Asp90. The catalytic cavity of CES1 is composed of two substrate-binding pockets: a small and rigid compartment (Leu96, Leu97, Leu100, Phe101, Leu358) which enables compound selectivity, and a large and flexible pocket (Thr252, Leu255, Leu304, Leu318, Leu363, Met364, Leu388, Met425, Phe426), which is promiscuous. This composition confers the ability to act on structurally diverse compounds. Figure 1 presents the human CES1 trimer, the active site composition and the binding modes of CES1 with the co-crystallized ligand naloxone (PDB ID 1MX9).

The catalytic triad residues, located between the two pockets, are aligned in a way that favors the generation of the Ser221 oxygen nucleophile. This nucleophile then attacks the carbonyl carbon of the ester substrate, leading to the formation of the acyl-enzyme intermediate, which is stabilized by the oxyanion hole residues Gly142 and Gly143. Additionally, the enzyme has two other ligand-binding sites referred to as the side-door and the Z-site: The side-door serves as an alternative/additional opening to the active site for substrate entrance or as a product release pore: The Z-site probably has an allosteric function by modulation of the binding site accessibility [45,46]. However, the side-door and the Z-site binding sites were not investigated in this study.

In the case of CES1, we have the luxury of 10 high-quality crystallographic structures representing a spectrum of pocket conformation, intuitively calling for an ensemble docking approach. Comparing the volume of the active site of the 10 X-ray structures, we noticed some differences going from 701 Å^3^ for 1YAH [19] to 1375 Å^3^ for 1MX9 [18], almost doubling the size (Figure S1 in Supplementary Materials). The solvent-accessible surface area (SASA) follows a similar trend with a SASA value two times higher for 1MX9 (356 Å^2^) than 1YAH (185 Å^2^) (Figure S2 in Supplementary Materials). This result shows large flexibility of the enzyme to adapt the accessibility of its binding site to large and diverse ligands and thus accurately represent different states of CES1 useful for applying ensemble docking. Therefore, we decided to assess the enrichment of an ensemble docking approach coupled with machine learning approaches to predict CES1 ligands.

#### 2.1.1. Evaluation and Validation of Docking Protocol

Redocking was conducted to assess the docking protocol for the given application: The co-crystallized ligands of eight of the 10 crystal structures were docked back in the defined binding pocket; and the conformation, orientation and position of the docked poses were compared to the experimentally determined ones. The two remaining structures (1YA8 and 2DQY) were crystallized with cleavage products located outside of the active site and could not be meaningfully redocked.

For ligands, homatropine, naloxone, ethyl acetate, benzyl, and palmitate, respectively, a root mean square deviation (RMSD) between 1.5 Å and 4.7 Å was observed with general conformations visually similar to their crystallized forms. Three notable outliers were the non-competitive inhibitor tamoxifen (RMSD around 8 Å) and the two ligands with many flexible bonds, taurocholate (RMSD around 6.7 Å) and coenzyme A (RMSD around 9 Å) (Figure S1 in Supplementary Materials). The relatively high RMSD can be explained by the dominance of non-specific hydrophobic interactions in ligand binding, with a very high average B-factor for the ligands themselves compared to the protein, indicating mobile ligands in the active site. A recent study has reported that at a resolution around 3 Angstrom, B-factors around 80 A² or higher indicate very low quality of the positional information for these atoms and that could explain our RMSD [47]. Furthermore, despite the high uncertainty over ligand position, the deposited PDB files do not contain alternate locations, an unfortunate choice that likely does not reflect the actual ligand-binding behaviour. Including alternate locations for the ligand would have likely reduced the average B-factor for each individual location [48].

While Autodock Vina may not be suitable for crystallographic pose prediction in this system, we considered the performance of Autodock Vina adequate for our purpose of screening inhibitors.

#### 2.1.2. Analysis of the Docking Protocol with Known CES1 Ligands

**Figure 2:**
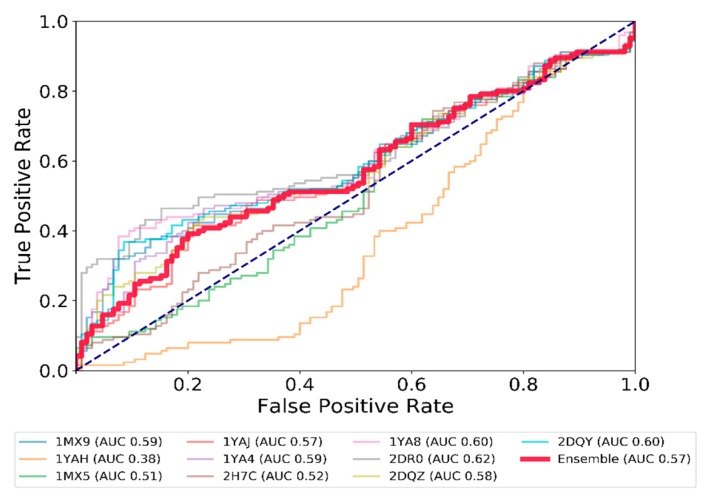
ROC curves showing the performance of docking based on the average energy score for each CES1 ligand docked in the 10 CES1 conformations. Each line corresponds to the results of the correct ranking of active ligands (with an activity below 10 µM) on one X-ray structure of CES1. The red line (Ensemble) considers the average energy score attributed to a ligand across every X-ray structure used in the docking protocol. The dashed line represents a random ranking.

Once the docking tool was validated, exhaustive docking for a set of 294 compounds with a known inhibition constant (Ki) at CES1 was completed with the 10 X-ray structures. To analyze the performance of the docking tool, the ROC curve (Receiver Operating Characteristic (ROC) was used, considering the Vina docking score and a 10 µM Ki threshold, splitting the dataset into an active and inactive group, with 140 and 154 ligands respectively. An ensemble docking approach was also considered, by taking the average of the scores of the 10 protein structures (Figure 2). The ROC curves were similar for seven conformations of the enzyme, showing a general discrimination ability of relatively weak size (AUC around 0.57–0.62). Two conformations remained close to the center line, with AUC 0.51 and 0.52, meaning that inactive compounds were docked in CES1 with a Vina docking score as good as for the active compounds. One structure (1YAH) showed inverse discriminative power (AUC 0.38), corresponding to the worst docking scores for the more active compounds. The ensemble docking (red line in Figure 2) does not show a better performance (AUC of 0.57) in comparison to many individual structures. Simple averaging of the score across all structure does not yield better performance. Seeking to explain such behavior, we noticed that 1YAH binds to the smallest substrate by far (ethyl acetate), with a corresponding small active site volume and exposed surface area. Small molecules like ethyl acetate were not found among the known high potency CES1 ligands in our dataset, thus high-scoring compounds on this protein structure were unlikely to be legitimate hits. Indeed, removing these false positives increased the discrimination ability of the other structures (elimination of the top 100 scoring compounds on the 1YAH structure allowed the AUC to go up by around 0.05 points for all other structures).

Interestingly, the ROC curves for most of the structures depict relatively good progress of the True Positive Rate (TPR) compared to the False Positive Rate (FPR) for the first 20% of the data set. Beyond the second decile, this metric does not allow prediction much better than chance. Exploring this finding further, we observed a markedly lower logP in best-scoring compounds (1st decile mean logP, 1.8; 10th decile, 4.6), as well as a lower molecular weight (mean 163 g/mol to 444 g/mol).

**Figure 3:**
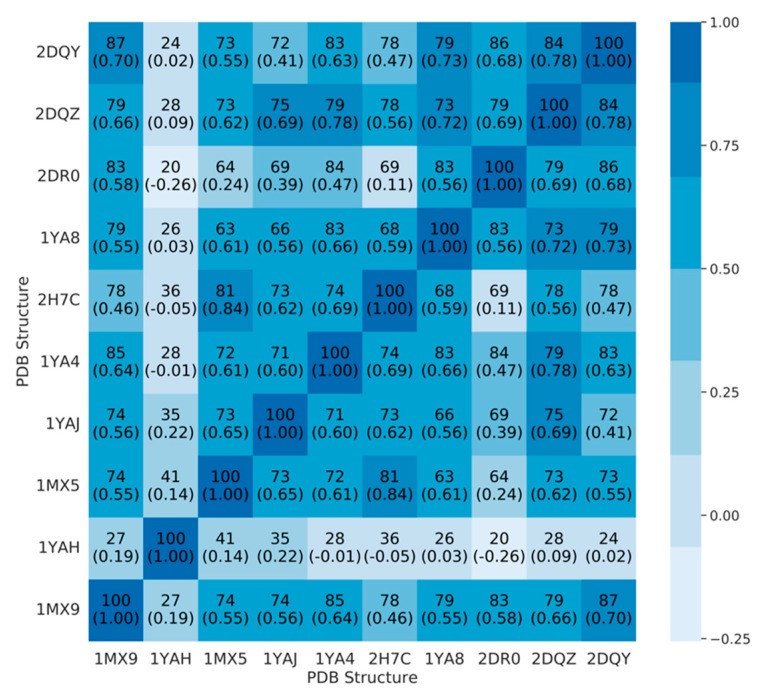
Heatmap showing the number of ligands in common in the top 100 scoring molecules for each pair of structures, and the Spearman R rank correlation for these ligands according to docking scores (in parenthesis). Color corresponds to Spearman R value.

To better understand the degree of (dis)agreement on ranking across structure, we computed a Spearman R rank correlation between the list of the 100 top scoring ligands, for each pair of CES1 structures (Figure 3). 

Except for the 1YAH structure, and to a lesser extent 2DR0, there is significant agreement between conformations on top scoring molecules, with 63% to 87% ligands found in both top 100, for each pair of structures. The ranking of these common ligands is also fairly consensual with a Spearman R coefficient in the 0.55–0.84 range. Two structures seem isolated from the rest, 1YAH and 2DR0, which depicted the worst and the best ROC curves with the Vina score. Additionally, there is local disagreement on compounds ranked (e.g., 1YAJ and 2DQY, 2DR0, and 2H7C), suggesting specific a binding mode of CES1. Each CES1 structure has between 12 and 51 active ligands in its top 100, three being common in any of the 10 structures (ChEMBL189162, ChEMBL242722, ChEMBL242932). It means that while there is significant pairwise overlap, a 10-structure ensemble is not equivalent to any one individual structure and is able to capture a more diverse set of ligands. When examining the top 100 worst scores of each structure, we find 35 to 46 active molecules, 33 of which are common to every structure (Table S1). These 33 ligands do not seem to be highly different to the remaining active ligands. The mean values for logP and molecular weight are also in the same range as the correctly predicted active compounds. The existence of a group of active yet badly scoring molecules, without obvious differences in chemical properties, suggests that docking score alone might not be sufficient to fully characterize ligand activity on CES1.

### 2.2. Expert System Based on Energy Terms

**Figure 4:**
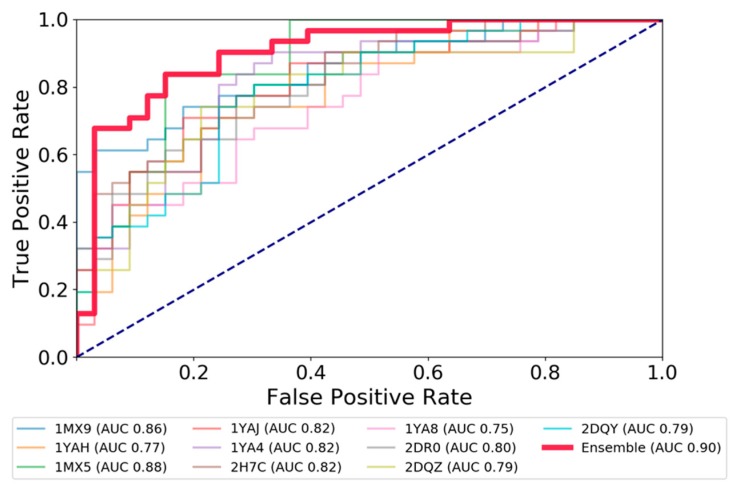
ROC curves showing the performance of the MLP classifier at a Ki threshold 10 µM, as measured on the test set.

Machine learning models using binding energy terms have shown to be an interesting alternative and complementary approach to predict the activity of ligands to a protein [49]. In Autodock Vina, selecting the “-score-only” option, we collected six individual terms calculated in the scoring function i.e., gauss1, gauss2, repulsion, hydrophobic, hydrogen interactions, and the Vina score (more information about these terms can be found in Ref. [50]). The values of each term for all the dockings were computed and averaged for each ligand and CES1 structure. In addition to structure-individualized input data, we evaluated an ensemble approach by taking the average of each component of the input vector for a given ligand across the 10 structures and resulting poses. The input data is available in Table S1. Then, three machine learning approaches (LDA, QDA and MLP) were developed using an arbitrary threshold of 1 µM, 10 µM and 100 µM to separate between active and inactive CES1 ligands. The performances of the models on a training set, cross validation and test sets are described in Table 1 and Figure 4. In general, all the models show a better AUC than the docking score only (between 10% and 20% higher). The AUC remains relatively high on the test set, demonstrating the robustness of the models. The best model was the neural network (MLP), followed by the QDA and LDA with a quite similar performance profile. In addition to cross-validation, to check for overfitting, the possibility of an outsized effect of liminal ligands was investigated by computing the performances of the classifiers while removing compounds with Ki within 5 µM of the 10 µM threshold from the dataset (i.e., removing compounds between 5 µM and 15 µM). For both procedures, there were minimal changes in the evaluation metrics (Figure S3), confirming a limited bias of compounds around the threshold in the models developed.

It confirms the benefit of combining machine learning approaches with ensemble models to improve the detection of CES1 ligands. Finally, we developed a MLP classification model using the entire set of 294 CES1 ligands and a threshold of 10 µM in order to investigate the CES activity of drugs. With a Matthews coefficient correlation of 0.74, the model shows a good performance and looks reliable for prediction.

### 2.3. Test and Validation of the Protocol to Predict CES1 Inhibitors

Based on the ensemble docking protocol, 1114 FDA-approved drugs were docked on the 10 X-ray CES1 structures, and the six terms of the scoring function were gathered. Then, using the ensemble model MLP trained previously on the full set of CES1 ligand, 679 FDA-approved drugs were predicted to be active on CES1 (Table S2). One of the most represented chemical classes was morphinan. In addition to the known ligand naloxone, a number of related drugs were rated likely CES1 inhibitors by our models (levallorphan, dextromethorphan, naltrexone, methadone, and meperidine among others). The tetracyclic and tricyclic antidepressants constitute a second family of top-rated potential CES1 inhibitors (maprotiline, nortriptyline, doxepin, maprotiline, mianserin among others). A few SSRIs/SNRIs were also found (atomoxetine, sertraline, fluvoxamine, duloxetine). Antidepressants with a documented inhibitory effect on CES1 include fluoxetine, thioridazine, and perphenazine [35]. Interestingly, the model predicted some steroids to be inhibitors of CES1, such as estriol, estradiol, testolactone, and testosterone. A handful of anti-muscarinic molecules were also found (procyclidine, tolterodine). The remaining hits were spread among a variety of pharmacological and chemical classes like benzodiazepine (zoldipem, clotiazepam, fludiazepam), antihistaminique (promethazine, trimeprazine), and local anesthetics (lidocaine, procaine, chloroprocaine, bupivacaine). Some of them were previously demonstrated to inhibit CES1, like diazepam [51].

**Figure 5:**
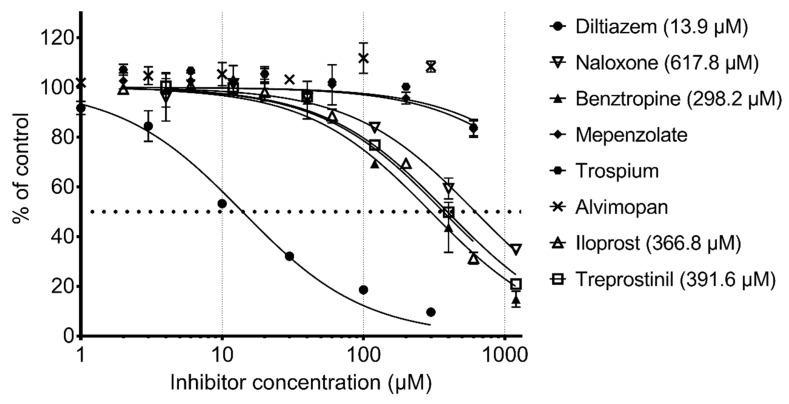
In-vitro assessment of CES1 activity inhibition by drugs.

To validate our model, we tested a set of eight molecules in an in-vitro assay including benztropine, naloxone, almivopan, iloprost, treprostinil, mepenzolate, trospium, and diltiazem (Figure 5). The selection of these was made upon consideration of different criteria, namely, the drug must be predicted as active as well as available and with different pharmacological action (Table 2). Nalbuphine was also selected, but since it lacked the required solubility in aqueous buffer at pH 7, we could not perform an experiment on this drug.

The positive control diltiazem displayed an IC50 of 13.9 µM in our assay. The known inhibitor naloxone was the less potent (617.8 µM). Almivopan exhibited no inhibition even at the highest concentration. Mepenzolate and trospium did not reach 50% inhibition in our assay (at max concentration 600 µM, they afforded ~15% and 30% inhibition, respectively). Benztropine was the second most potent inhibitor of the predicted compounds with an IC50 value of 298.2 µM, followed by iloprost, which exhibited an IC50 of 366.8 µM and treprostinil with an IC50 of 391.6 µM. Benztropine has previously been tested on CES1, but no IC50 value was indicated in the study [35]. We can show here that benztropine is a weak inhibitor.

## 3. Discussion

Drug interactions are recognized as a significant factor contributing to the occurrence of ADRs and their number increases as more drugs are released into the market. There are different mechanisms by which drug interactions can occur, for instance, in pharmacokinetics-related mechanisms where drug absorption, distribution, metabolism and/or excretion are affected; or pharmacodynamics-related mechanisms, when drugs with similar pharmacological actions are co-prescribed. Knowing the mechanisms involved in drug interactions is essential to be able to predict and prevent them. CES1 is the major hydrolytic enzyme responsible for the biotransformation of many and diverse therapeutic agents including Angiotensin-converting-enzyme inhibitors, anti-cancer prodrugs, narcotic drugs, methylphenidate, and others. Significant interindividual variability of pharmacokinetic and pharmacodynamic profiles of these medications, often associated with unforeseen ADRs and therapeutic failure, has been frequently observed in clinical practice. The main underlying causes are genetic variation and DDIs [38]. Previously published studies [35,52] have identified some classes of routinely prescribed medications as potential inhibitors of CES1, however, none of these studies performed a systematic screening of all clinically-used drugs. Hence, in the present study, we sought to focus on potential DDI resulting from CES1-mediated metabolism inhibition by clinically used drugs, using a methodology combining ensemble docking and machine learning approaches. This protocol allows for an evaluation of large databases of compounds from which only a limited number of compounds can be selected and experimentally evaluated. 

Thanks to the availability of many crystal structures of CES1–ligand complexes, docking simulations were carried out to unravel molecular-level mechanisms of drug binding potentially producing inhibitory effects of CES1. Furthermore, using the energies terms calculated in the Vina score, MLP, LDA and QDA models were performed, improving the prediction of new CES1 ligands.

Using our method on DrugBank, we identified a range of different therapeutic agents as potential CES1 inhibitors including several commonly used agents such as estrogens and fluoxetine. Importantly, several of the drugs, which we found to be potential inhibitors of CES1 by our in-silico approach, have previously been shown to inhibit CES1 in vitro. This includes maprotiline, nortriptyline, amitriptyline, fluoxetine, sertraline, and estriol. In particular, the SSRI sertraline has been found to have a strong in-vitro inhibitory effect on the activity of CES1 [35,53]. Overall, our prediction that many diverse steroids have an inhibitory effect on CES1 is in line with published in-vitro findings, which have suggested that progesterone, the progesterone metabolite 5β-pregnan-3,20-dione, testosterone, corticosterone, and deoxycorticosterone, in addition to estrogens, inhibit CES1 [53].

These results demonstrate the usefulness of such approach in validating known biologically active drugs, but also in identifying new drugs which potentially affect the enzyme. However, the many and structurally diverse compounds recognized by CES1 lead to the likelihood of numerous binding modes of inhibitors and substrates and so it can affect the scoring docking poses. Applying a machine learning approach using the different energies terms from Vina has the advantage to fit the mathematical model to the previous knowledge data considered (training set) and so to make a decision for the new compounds investigated. There are other pitfalls, which make the prediction of binding conformations difficult like the low resolution of crystal structures and more especially conformational changes that occur upon binding. Receptor flexibility remains a challenge in docking simulations. Various approaches such as soft-docking strategies, protein side-chain rotamer libraries, ensemble-based docking, molecular dynamics, and Monte Carlo simulations are able to incorporate some degree of receptor flexibility into the docking process [54]. Furthermore, the presence of the so called ‘activity cliffs’, i.e., pairs of structurally similar compounds having a significantly different biological activity can negatively impact the performance of the similarity searching. Finally, in-vitro testing of the predicted hits can also be problematic, mainly due to poor water solubility of compounds. All these limitations have to be considered when such a computational study is developed.

## 4. Materials and Methods

### 4.1. Collection and Curation of the Chemical Library

One thousand one hundred and fourteen FDA-approved, non-nutraceutical drugs were collected from Drugbank 4.0 [55] and a reference dataset of 294 ligands with biological activity on CES1 (documented Ki) was gathered from Chembl [56]. Conformers for those molecules were either retrieved through PubChem [57], returning the experimental structure conformer if available, or generated using the open source cheminformatics library, RDKit [58]. 

Ligands were converted to PDBQT format using Autodock Tools (Scripps Research Institute), assigning Gasteiger charges to the atoms at the same time [59]. pH-appropriate protonation was conducted using OpenBabel (pH set at 7.4) [60]. The molecules were then randomly assigned to a training set of 230 compounds (80%) and a test set of 64 compounds (20%) for use in the development and validation of the predictive models. All Tanimoto similarity distances were computed based on Daylight fingerprint, as implemented in RDKit.

### 4.2. Protein Preparation and Docking Protocol

The coordinates of the 10 CES1 complexed with different non-covalent ligands were obtained from the RCSB PDB [61]. The pdb files used in this study are 1MX9, 1YAH, 1MX5, 1YAJ, 1YA4, 2H7C, 1YA8, 2DR0, 2DQZ, and 2DQY. For each structure, using UCSF Chimera [62], only the A chain was kept, from which water and molecules other than amino acid residues were removed. The Dock Prep utility was used on the protein for protonation (residue-name based for HIS, GLU, ASP, LYS, and CYS), assigning charges (using AMBER ff14SB for standard residue, Gasteiger charges otherwise). The structures were aligned and the amino acid renumbered to ensure direct comparability of docking results. Active site pocket metrics were assessed using fpocket 3.0 [63], with default parameters except for a clustering distance of 2.8 angstrom to obtain a single cluster encompassing the active site in all 10 structures. This setting slightly overestimates pocket volume in absolute value but allows for a reliable comparison between the structures

### 4.3. Docking Protocol 

Docking was performed using Autodock Vina [59]. A single docking box centered around the mechanistically-relevant Ser 221 was used, with a size 26 × 28 × 32 Angstrom for the 10 receptor structures. The exhaustiveness parameter, which corresponds to the amount of sampling effort, was set to eight, and the desired number of poses to three. Docking scores distribution was examined for irregularities or outliers. The distance from the ligand centroid to the catalytically important Ser 221 was taken as evidence of the ligand being docked into the active site. For Vina, a large majority of poses were less than 10 Å from this serine, with a set of outliers at 55 Å.

### 4.4. Machine Learning Models

Using the python library SciKit-Learn and Keras [64] (using TensorFlow as backend), three types of machine learning models i.e., Linear Discriminant Analysis (LDA), Quadratic Linear Discriminant analysis (QDA) and a MultiLayer Perceptron (MLP) were trained to classify active and inactive ligands using the ChEMBL data. Three thresholds (1 µM, 10 µM, and 100 µM) were used to discriminate between the two classes (active and inactive ligands). The input data on the models consisted of the score and five docking terms from the Vina’s score_only mode (gauss1, gauss2, repulsion, hydrophobic, and hydrogen interactions), all extracted from each pose of the ChEMBL data. One aspect of the score, namely the entropic term related to the number of rotatable bonds, was not extractable using score_only mode. However, it is represented in the Vina score itself. The input data were normalized to mean 0 and variance 1 as a preprocessing step. The necessary scaling was determined on the training data and used for both training and test data

The multilayer perceptron (MLP) was set up as follows: One input layer of six nodes, two hidden layers of 16 nodes with sigmoid activation function, and one output layer of one node using the sigmoid activation function. Batch renormalization was done between the two hidden layers and before the output layer. The optimizer used was RMSProp, the loss function was binary cross-entropy, and the batch size was 64. Training was run for 2000 epochs. 

Two kind of models were trained. In the 10 structure-individualized models, the input data generated by docking on a given structure was used. The values of the six components for a given ligand were obtained by averaging over the three generated poses. On the other hand, in the ensemble model, the data generated by every structure were considered (using the average overall generated poses). The models were trained on a training set of 230 ligands (80%) and validated on a test set of 64 ligands (20%), which were randomly selected. A five-fold cross validation was performed on the training set. The performance and robustness of the models were assessed using the AUC, and Matthews coefficient correlation on the test set.

### 4.5. In Vitro Assessment of CES1 Inhibition 

Benztropine mesylate, mepenzolate bromide, and trospium chloride were ordered at Sigma-Aldrich (St. Louis, MO, US). Alvimopan was from Toronto Research Chemicals (Toronto, ON, Canada). Iloprost and treprostinil were from Cayman Chemical (Ann Arbor, MI, US). Diltiazem hydrochloride was from Napp Pharmaceuticals Research (Cambridge, UK). Naloxone was from LGC Standards (Middlesex, UK). Recombinant human CES1 was from BD Gentest (Woburn, MA, US). Inhibition of CES1 by these compounds was investigated by incubation with the recombinant enzyme in the presence of the ester substrate *p*-nitrophenyl acetate. The concentration of hydrolytic product, *p*-nitrophenol, was determined by measurement of absorbance at 405 nm after 3 min with a Sunrise microplate reader (Tecan, Grödig, Austria). The incubations were performed in 96-well pureGrade BRANDplates (BRAND, Wertheim, Germany) in 100 mM phosphate buffer at 37 °C in a final volume of 200 mL. Dimethyl sulfoxide (DMSO) was used for the dissolution of all compounds. The final concentration of DMSO was 2% v/v in all incubations including the controls. This concentration was previously found to have a negligible effect on enzyme activity [51]. The substrate and inhibitor were premixed to allow the simultaneous addition of both. The final concentration of substrate was 100 µM, and the final protein concentration was 10 µg/mL. The potential inhibitors were incubated at six concentration levels in the range 1–1200 µM. The sampling time was tested to be in the linear range of the reaction, and the drugs were tested in triplicates. Substrate in buffer without enzyme was included as a negative control. The results were corrected for spontaneous hydrolysis by subtracting the absorbance of the negative control and comparing with a positive control containing enzyme, but no inhibitor. IC_50_ constants were determined for compounds displaying significant inhibition using non-linear regression in Prism, version 6.07 (GraphPad Software, Inc., San Diego, CA, USA).

## 5. Conclusions

Overall, we have demonstrated that a methodology combining ensemble docking and machine learning methods is a valuable tool for the initial identification of potential new CES1 ligands. After experimental tests, some of our hits were proven to be novel CES1 inhibitors. Although only modest inhibitors, they might be pharmacokinetically important in scenarios involving drugs with narrow therapeutic margins. The clinical relevance of these finding should be investigated further.

Beyond CES1, our in-silico techniques may be applicable more generally to virtual screening for enzymes with broad substrate selectivity.

## Figures and Tables

**Table 1:** Performance of the ensemble models (including the non-machine-learning score-only classifier) under different activity thresholds and machine learning approaches.

Threshold	1 µM	10 µM	100 µM
**Number of active ligands**			
of the 230 in the training set	76 (33%)	107 (46%)	127 (55%)
of the 64 in the test set	19 (30%)	33 (52%)	36 (56%)
**Vina score (no statistical model)**			
Ensemble AUC (training set)	0.54	0.57	0.63
Best AUC among all individual structures	0.58	0.62	0.66
**Ensemble MLP model**			
AUC (std. dev.) 5CV (training set)	0.84 (0.05)	0.81 (0.03)	0.75 (0.06)
AUC (test set)	0.82	0.90	0.92
Matthews coeff. (test set)	0.49	0.56	0.59
**Ensemble LDA model**			
AUC (std. dev.) 5CV (training set)	0.78 (0.10)	0.77 (0.08)	0.81 (0.07)
AUC (test set)	0.77	0.77	0.77
Matthews coeff. (test set)	0.38	0.34	0.43
**Ensemble QDA model**			
AUC (std. dev.) 5CV (training set)	0.79 (0.12)	0.77 (0.08)	0.81 (0.10)
AUC (test set)	0.76	0.78	0.75
Matthews coeff. (test set)	0.31	0.47	0.39

**Table 2:** Name, 2D structure, IC50, and pharmacological action of the eight drugs tested in vitro on a CES1 assay. NS means Non-significant.

Name	2D Structure	IC50 (µM)	Pharmacological Action
Diltiazem	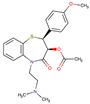	13.9	Cardiovascular diseases. Antihypertensive and vasodilating properties.
Naloxone	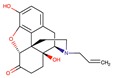	617.8	Indicated for the rapid reversal of symptoms of central nervous system depression in opioid overdose.
Benztropine	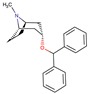	298.2	Treatment of Parkinson’s disease.
Mepenzolate	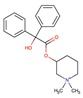	NS	Gastrointestinal disorders. Decrease gastric acid and pepsin secretion.
Iloprost	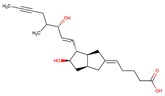	366.8	Pulmonary arterial hypertension.
Trospium	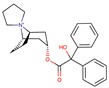	NS	Antispasmodic agent.
Alvimopan	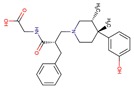	NS	Drug for constipation. Accelerates the gastrointestinal recovery after bowel surgery.
Treprostinil	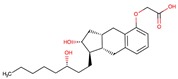	391.6	Pulmonary arterial hypertension.

## Supplementary Materials

Supplementary materials are available online https://www.mdpi.com/1420-3049/24/15/2747/s1.
