# Supplementary material for: Combined Ensemble Docking and Machine Learning in Identification of Therapeutic Agents with Potential Inhibitory Effect on Human CES1
Source: Molecules. 2019 Jul 29;24(15):2747. doi: 10.3390/molecules24152747 (PMC6696021; doi:10.3390/molecules24152747)
Supplement: Supplementary file 1 [file molecules-24-02747-s001.pdf]

Supplementary information

Molecules repartition into the various groups

- 296 ligands with documented Ki from ChEMBL, from which:
  - 232 ligands assigned to the training group (80%)
  - 64 ligands assigned to the testing group (20 %)

- For all docked ligands:
  - 3 poses produced

- Drugbank: 6631 molecules downloaded
  - 148 could not be parsed by RDKit
  - 232 molecules that could not be docked by Vina (eg: all molecules with inorganic atoms)
  - 5078 non-FDA approved molecules, not used (eg: experimental drugs, illicit drugs)
  - 291 FDA approved nutraceuticals, not used (eg: vitamin C, tetrahydrofolate)
  - **1114 FDA approved drugs, used in the analysis.**

Redocking of the co-crystallised ligands of the PDB structures

**CES1 structure**	1MX5	1MX9	1YA4	1YA8	1YAH
**Ligand**	Homatropine	Naloxone	Tamoxifen	Mevastatin	Ethyl acetate
**Ligand 2D structure**	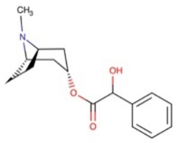	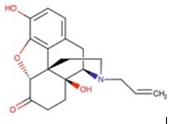	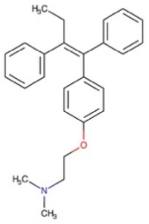	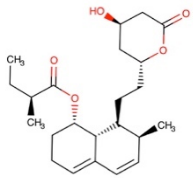	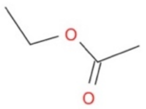
Redocking applicable?	Yes	Yes	Yes	No, cleavage products	Yes
**RMSD** between crystalized ligands and the best docked pose (Angstrom range on the best poses)	1.5	4.5	7.2	N/A	4.7
**Score of best pose** (~estimated Gibbs energy of binding in kcal/mol)	-8.4	-7.9	-8.2	-8.0	-3.6

**Structure**	1YAJ	2DQY	2DQZ	2DR0	2H7C
**Ligand**	Benzil	Cholate<br>Palmitate	Homatropine<br>Coenzyme A<br>Palmitate	Taurocholate	Coenzyme A
**Ligand 2D structure**	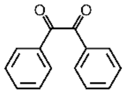	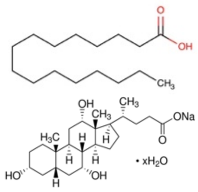	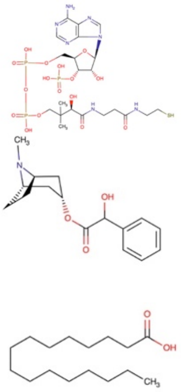	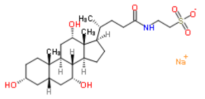	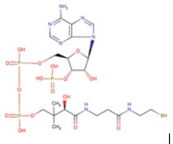
Redocking applicable?	Yes	No<br>(outside active site)	Yes<br>(Palmitate)	Yes	Yes
**RMSD** between crystalized ligands and the best docked pose (Angstrom range on the best poses)	3.5	N/A	3.7	6.5	8.7
**Vina score of best pose** (~estimated Gibbs energy of binding in kcal/mol)	-5.2	-8.0	-5.3	-9.2	-7.2

Figure S1: Tabulated results of the redocking of the co-crystallised ligand of each PDB structure, done on Autodock Vina with the same settings as in the main matter.

Active site characterization according to fpocket 3.0

Default settings except for clustering distance (2.8 angstrom), leading to slightly overestimated volume and SASA, but yielding one and only one detected pocket covering the active site, allowing for structure-to-structure comparisons. (Addition of the value of multiple pocket is non-ideal for comparison purpose)

Structure	1YAH	1YAJ	1YA8	2DQZ	1YA4	2H7C	1MX5	2DR0	2DQY	1MX9
**Volume** (active site, A^3)	**701**	**795**	**950**	**958**	**984**	**1015**	**1071**	**1078**	**1157**	**1375**
**Total SASA**	**185**	**189**	**227**	**224**	**222**	**274**	**276**	**258**	**269**	**356**
Polar SASA	43	34	59	60	72	98	85	71	71	103
Apolar SASA	142	155	168	164	150	176	191	187	198	253

Figure S2: Active site volume and SASA for each structure, according to fpocket

	Complete dataset except ligands with Ki between 5 and 15 μM	Complete dataset
**Threshold**	**10 μM**	**10 μM**
**Number of active ligands**	135 (50%)	140 (47%)
**Vina score only (no statistical model)**<br>Ensemble AUC (complete set)<br>Best AUC among all individual structures	 0.55<br>0.60	 0.57<br>0.62
**Ensemble MLP model**<br>AUC (std. dev.) 5CV (training set)	0.83 (0.02)	0.81 (0.03)
**Ensemble LDA model**<br>AUC (std. dev.) 5CV (training set)	0.77 (0.07)	0.77 (0.08)
**Ensemble QDA model**<br>AUC (std. dev.) 5CV (training set)	0.77 (0.06)	0.77 (0.08)

Figure S3: Comparison of the MLP, QDA and LDA models removing ligands with an activity between 5 μM and 15 μM.

Table S1: 294 compounds with known activity on CES1 gathered in ChEMBL database.

For each chemical, the ChEMBL ID, the SMILES code, the bioactivity (Ki) and the energy scores from autodock vina are depicted.

Set	Name	SMILES	Ki_Nanomolar	Vina_Score_Best	Vina_Comp_Gauss1	Vina_Comp_Gauss2	Vina_Comp_Repulsion	Vina_Comp_Hydrophobic	Vina_Comp_Hydrogen
Training	CHEMBL467451	CCCCCCCCCCS(=O)(=O)CC(O)(O)C(F)(F)F	397,32	-7,47775	80,56594	1366,81469	2,06895	72,65263	3,40717
Training	CHEMBL463556	CCCCCCCCCCCC[S+]([O-])CC(O)(O)C(F)(F)F	952,93	-7,22684	86,86914	1410,26186	1,90733	76,3468	2,9057
Training	CHEMBL510996	OC(O)(CSCCc1ccccc1)C(F)(F)F	6904,93	-7,81929	70,02355	1062,41055	2,21021	66,53035	3,60409
Training	CHEMBL270375	CCCCCCCCCCCCSCC(=O)C(F)(F)F	39,1	-6,75294	78,59238	1311,69721	0,93567	83,72162	0,96949
Training	BENZIL	O=C(C(=O)c1ccccc1)c2ccccc2	43,89	-8,41449	58,31861	1034,93222	0,59384	76,17376	0,52242
Training	CHEMBL192180	Clc1ccc(cc1)C(=O)C(=O)c2ccccc2	37,02	-8,5064	57,27003	1108,01636	1,24201	55,49481	2,29183
Training	CHEMBL235287	O=C(C1CCCCC1)C(=O)C2CCCCC2	38,5	-8,16161	50,65656	1038,95781	1,51916	59,57889	2,75868
Training	CHEMBL242067	Fc1cccc(c1)C(=O)C(=O)c2cccc(F)c2	100	-8,98422	65,46624	1144,72568	0,61748	71,9835	0,55112
Training	CHEMBL192139	Clc1ccc(cc1)C(=O)C(=O)c2ccc(Cl)cc2	25091,39	-8,67776	64,22728	1161,60784	1,50946	58,41334	1,94546
Training	CHEMBL244428	OC(C(=O)c1cc(F)ccc1F)c2cc(F)ccc2F	230	-9,32485	66,12775	1270,59123	0,58902	66,91098	0,34716
Training	CHEMBL189162	O=C(C(=O)c1ccc2ccccc2c1)c3ccc4ccccc4c3	256,02	-10,84852	81,15214	1417,1904	1,34103	104,93531	0
Training	CHEMBL242721	Fc1ccc(F)c(c1)C(=O)C(=O)c2cc(F)ccc2F	260	-9,62906	72,87742	1268,83609	0,64302	69,15322	0,50993
Training	CHEMBL191847	Cc1ccc(cc1[N+](=O)[O-])C(=O)C(=O)c2ccccc2	223,16	-9,23514	63,40987	1305,30756	0,81874	65,4579	1,35734
Training	CHEMBL193180	Fc1cc(cc(F)c1F)C(=O)C(=O)c2cc(F)c(F)c(F)c2	404,51	-9,53344	75,40766	1388,10758	1,48017	63,94381	0,61831
Training	CHEMBL370059	OC(=O)c1ccc(cc1)C(=O)C(=O)c2ccccc2	524,4	-8,73997	63,73287	1233,38111	1,29263	55,47093	2,20684
Training	CHEMBL244630	OC(C(=O)c1cc(F)cc(F)c1F)c2cc(F)cc(F)c2F	665	-10,07944	78,50125	1399,58339	0,93291	66,05757	0,51108
Training	CHEMBL365445	O=C(C(=O)c1cccs1)c2cccs2	433,27	-6,42133	49,78783	908,32178	1,03147	18,61164	2,22339
Training	CHEMBL385800	Clc1cc(Cl)c2C(=O)C(=O)Nc2c1	890	-7,32199	47,89912	837,39539	1,05257	26,21497	2,15351
Training	CHEMBL1812860	CCC(=O)C(=O)c1ccccc1	1550	-6,6494	53,60472	770,26078	1,16872	54,84306	1,69809
Training	CHEMBL426562	CCc1ccc(NCN2C(=O)C(=O)c3ccccc23)cc1	1880	-9,41329	64,83756	1374,95911	0,53909	76,21014	0
Training	MILTIRONE	CC(C)C1=Cc2ccc3c(CCCC3(C)C)c2C(=O)C1=O	2530	-10,06835	79,77882	1330,62365	3,57133	102,63468	0,61589
Training	BENZOIN	OC(C(=O)c1ccccc1)c2ccccc2	30427,5	-8,33978	57,42424	1037,27893	0,68385	76,59549	0,50589
Training	CHEMBL483675	Fc1ccccc1S(=O)(=O)Nc2ccc(NS(=O)(=O)c3ccccc3F)cc2	100000	-9,89052	77,59333	1657,32655	1,49485	51,18964	2,57756
Training	CHEMBL488111	Cc1ccc(cc1S(=O)(=O)Nc2ccc(NS(=O)(=O)c3ccc(F)cc3)cc2)C(=O)O	100000	-10,50634	102,85747	1816,02934	1,35154	71,54646	0,69414
Training	CHEMBL1630815	COc1ccc(CC(=O)Nc2cccc(c2)C(=O)C(=O)c3ccccn3)cc1	100000	-9,73752	107,7691	1675,52374	1,83414	71,85072	0,46866
Training	CHEMBL374629	CCc1ccc2C(=O)C(=O)Nc2c1	100000	-7,60543	52,85089	835,81785	1,04775	42,60136	2,11795
Training	CHEMBL365392	Clc1ccccc1C(=O)C(=O)c2ccccc2Cl	1654,8	-9,01684	63,8647	1170,18792	1,32945	58,52189	2,31305
Training	CHEMBL191796	[O-][N+](=O)c1cccc(c1)C(=O)C(=O)c2cccc(c2)[N+](=O)[O-]	18148,5	-9,23545	79,75021	1398,18531	1,26035	64,12306	1,19307
Training	CHEMBL222989	OC(=O)CN1C(=O)C(=O)c2ccccc12	100000	-7,80395	52,66948	949,03545	0,71657	38,51236	2,04793
Training	CHEMBL1812848	O=C(Sc1ccccc1)C(=O)Sc2ccccc2	100000	-8,35736	65,47037	1156,99743	1,21231	64,28304	2,1602
Training	BENZYLSULFONYLBENZENE	O=S(=O)(Cc1ccccc1)c2ccccc2	100000	-8,12814	54,26017	1037,69486	0,57479	71,01292	0,44732
Training	CHEMBL192657	Oc1ccc(cc1[N+](=O)[O-])C(=O)C(=O)c2ccc(O)c(c2)[N+](=O)[O-]	100000	-8,74837	86,56156	1538,02518	2,78062	42,35277	1,94425
Training	CHEMBL372856	COc1ccc(cc1[N+](=O)[O-])C(=O)C(=O)c2ccc(OC)c(c2)[N+](=O)[O-]	100000	-8,48328	78,85954	1553,99731	2,27939	25,23942	3,69167
Training	CHEMBL3526991	COc1ccc2cc(ccc2c1)[C@H](C)C(=O)O[C@@H]3O[C@@H]([C@@H](O)[C@H](O)[C@H]3O)C(=O)O	100000	-10,55628	103,81506	1850,05637	4,34529	70,92408	3,73853
Training	CHEMBL362996	O=C(C(=O)c1ccc(cc1)C(=O)C(=O)c2ccccc2)c3ccccc3	6,51	-10,01918	95,1292	1602,71353	2,92686	74,43173	2,9442
Training	CHEMBL242931	Fc1ccc(C(=O)C(=O)c2ccc(F)c(F)c2F)c(F)c1F	91,3	-9,79559	70,88177	1400,95868	0,86036	62,71961	0,5034
Training	CHEMBL192252	Cc1ccc(cc1)C(=O)C(=O)c2ccc(Cl)cc2	159,25	-8,98502	64,95234	1160,95529	1,47397	68,29297	1,88405
Training	CHEMBL435109	COc1cccc(c1)C(=O)C(=O)c2cccc(OC)c2	1544,41	-8,66235	72,47659	1288,08736	0,60559	61,38337	0,56011
Training	CHEMBL373781	O=C1N(CCN2C(=O)C(=O)c3ccccc23)c4ccccc4C1=O	1560	-9,95806	78,78486	1393,09695	1,87989	61,86031	1,92384
Training	CHEMBL115581	O=C1N(Cc2ccccc2)c3ccccc3C1=O	1700	-9,14486	56,37341	1184,03787	1,47356	62,96093	1,91877
Training	CHEMBL385799	Clc1ccc2NC(=O)C(=O)c2c1Cl	3020	-7,23284	54,03391	829,06956	1,32533	26,60166	2,0681
Training	CHEMBL376435	FC(F)(F)c1cccc2C(=O)C(=O)Nc12	13100	-7,99762	51,33435	948,63832	1,57669	43,97284	2,60552
Training	CHEMBL364734	COC(OC)(C(=O)c1ccccc1)c2ccccc2	15300	-7,84272	65,00928	1227,58869	1,74887	72,4431	0,7175
Training	CHEMBL221669	Cc1c(Cl)ccc2C(=O)C(=O)Nc12	55300	-7,53289	48,81215	825,52685	1,19461	35,19126	2,22789
Training	CHEMBL224884	Cc1cccc2C(=O)C(=O)Nc12	100000	-7,14436	42,45255	774,55561	1,04012	34,4811	2,2219
Training	CHEMBL235490	COc1ccccc1C(=O)C(=O)c2ccccc2OC	100000	-8,23816	71,06835	1303,63756	1,80145	46,12607	2,19823
Training	CHEMBL1812847	O=C(Nc1ccccc1)C(=O)Nc2ccccc2	100000	-9,28367	65,9747	1168,43156	0,88983	69,75292	1,42933
Training	CHEMBL482847	Brc1cccc(c1)S(=O)(=O)Nc2ccc(NS(=O)(=O)c3cccc(Br)c3)cc2	100000	-9,83038	88,82605	1611,6063	1,49041	67,38168	1,18791
Training	CHEMBL486277	Clc1ccc(cc1)S(=O)(=O)Nc2ccc3c(Cc4cc(NS(=O)(=O)c5ccc(Cl)cc5)ccc34)c2	100000	-11,91805	129,99924	2040,4263	2,86073	81,55372	0,8334
Training	CHEMBL371371	CC1CCC(=O)C1=O	100000	-5,50984	50,95463	616,23194	1,60807	32,09282	1,26846
Training	CHEMBL193228	Brc1ccc(cc1)C(=O)C(=O)c2ccc(Br)cc2	66666,68	-8,547	70,18543	1154,46656	1,88779	59,97735	1,84487
Training	CHEMBL1812851	BrC(C(=O)C(=O)C(Br)c1ccccc1)c2ccccc2	107	-9,37806	72,69869	1298,11143	1,35032	90,68415	1,35575
Training	CHEMBL397739	Fc1ccc(cc1F)C(=O)C(=O)c2ccc(F)c(F)c2	300	-9,30993	66,245	1276,15007	0,5054	63,98799	0,31271
Training	CHEMBL402615	CCCCSCC(=O)C(F)(F)F	399,85	-5,83516	59,03436	762,61043	0,98716	41,0578	2,10881
Training	CHEMBL222149	CC(C)(C)c1ccc(NCN2C(=O)C(=O)c3ccccc23)cc1	1420	-9,92818	92,59649	1456,479	4,1703	98,63198	2,52753
Training	CHEMBL191513	COc1ccc(cc1)C(=O)C(=O)c2ccc(OC)cc2	3399,22	-8,29908	72,92483	1276,22205	0,81115	59,16641	0,27403
Training	CHEMBL60569	CN1C(=O)C(=O)c2ccccc12	5380	-6,89867	38,37975	793,31942	0,98437	26,55915	2,27521
Training	CHEMBL236340	[O-][N+](=O)c1ccc(cc1)C(=O)C(=O)c2ccc(cc2)[N+](=O)[O-]	9800	-8,42342	74,66006	1383,34614	1,04858	56,48113	0
Training	CHEMBL3526940	O[C@@H]1[C@@H](O)[C@H](OC(=O)[C@@H](N2CCc3sccc3C2)c4ccccc4Cl)O[C@@H]([C@H]1O)C(=O)	24800	-10,02427	106,85155	1966,8423	4,79671	52,57531	3,04933
Training	CHEMBL521447	Clc1cc(Cl)cc(c1)S(=O)(=O)Nc2ccc(NS(=O)(=O)c3cc(Cl)cc(Cl)c3)cc2	100000	-9,60194	99,40746	1788,37903	3,56744	62,01025	1,76515
Training	CHEMBL482442	Clc1cc(Cl)c(cc1Cl)S(=O)(=O)Nc2ccc(NS(=O)(=O)c3cc(Cl)c(Cl)cc3Cl)cc2	100000	-10,50645	98,52137	1858,03894	2,12142	61,18551	1,26792
Training	CHEMBL485244	Fc1ccc(cc1)S(=O)(=O)Nc2ccc(NS(=O)(=O)c3ccc(Cl)cc3)cc2	100000	-10,02776	87,96037	1677,28653	1,52369	62,81881	1,42913
Training	CHEMBL449775	CCCCCCS(=O)(=O)CC(O)(O)C(F)(F)F	4886,54	-7,34369	64,88856	1131,43965	1,39772	50,56615	3,46836
Training	CHEMBL1812855	CCCCC(=O)C(=O)CCCC	46,6	-5,66361	52,06307	783,45143	0,8759	49,05707	1,88083
Training	CHEMBL193229	Fc1cc(F)cc(c1)C(=O)C(=O)c2cc(F)cc(F)c2	79,71	-9,4956	71,4547	1269,14506	0,74051	68,98809	0,46748
Training	CHEMBL193140	Cc1ccc(cc1C)C(=O)C(=O)c2ccccc2	79,25	-9,33742	68,81555	1159,92548	1,18911	89,65218	0,68744
Training	CHEMBL244425	OC(C(=O)c1ccc(F)cc1F)c2ccc(F)cc2F	480	-9,37601	66,00597	1284,92588	0,66256	65,60037	0,52048
Training	CHEMBL385132	Brc1ccc(NCN2C(=O)C(=O)c3ccccc23)cc1	580	-8,91028	73,55178	1287,58828	3,31125	68,77809	2,70745
Training	CHEMBL395654	O=C1N2CCCc3cccc(C1=O)c23	1540	-7,93087	47,04348	900,66853	1,14621	43,62743	1,77756
Training	CHEMBL116649	FC(F)(F)Oc1ccc2NC(=O)C(=O)c2c1	16500	-8,2954	60,28244	1019,10747	1,17516	35,78179	2,72358
Training	CHEMBL374758	Brc1ccccc1NCN2C(=O)C(=O)c3ccccc23	36000	-9,40267	63,5697	1312,60911	1,49429	65,87584	1,65189
Training	CHEMBL191800	CC1CC(C)(C)CC(=O)C1=O	100000	-6,56982	36,27535	721,66588	1,17798	33,74886	2,31976
Training	CHEMBL519072	Cc1c(C)c(NS(=O)(=O)c2ccc(F)cc2)c(C)c(C)c1NS(=O)(=O)c3ccc(F)cc3	100000	-9,91833	91,93335	1785,65773	2,08132	75,80266	0
Training	CHEMBL499975	CCOc1ccc(NS(=O)(=O)c2ccc(Cl)cc2)cc1	100000	-8,46427	72,86753	1284,8476	1,38525	58,70453	1,40017
Training	CHEMBL484276	Clc1ccc(cc1)S(=O)(=O)Nc2ccc(NS(=O)(=O)c3ccc(Cl)cc3)c4ccccc24	100000	-11,26845	116,96891	1876,36195	2,84522	94,5938	0,78027
Training	CHEMBL488335	Cc1cc(C)c(NS(=O)(=O)c2ccccc2)cc1NS(=O)(=O)c3ccccc3	100000	-10,00144	96,46826	1637,26802	2,61626	78,69576	1,83297
Training	CHEMBL442046	CCc1cccc2NC(=O)C(=O)c12	100000	-7,16524	49,90631	841,8815	1,05021	33,1835	2,01745
Training	CHEMBL364893	OC(C(=O)c1occc1)c2occc2	100000	-6,88999	49,43846	849,3804	1,43014	22,99156	4,00965
Training	CHEMBL370299	COc1ccc(cc1)C(O)C(=O)c2ccc(OC)cc2	100000	-8,269	72,3837	1299,12047	0,85876	57,75723	0,18588
Training	CHEMBL235288	O=C1Cc2ccccc2C1=O	5240	-6,84184	39,4452	710,65521	0,65077	37,19625	1,72838
Training	CHEMBL192258	CC(=O)C(=O)c1ccccc1	5270	-6,49337	47,76404	709,33133	0,73807	48,1268	1,41012
Training	CHEMBL371181	O=C(C(=O)c1occc1)c2occc2	3608,3	-6,70348	53,19953	915,52786	1,83981	20,73648	3,54592
Training	DIBENZOYLMETHANE	O=C(CC(=O)c1ccccc1)c2ccccc2	26000	-8,66844	58,2215	1116,06524	0,95128	80,94825	1,40798
Training	CHEMBL460808	CCCCCC[S+]([O-])CC(O)(O)C(F)(F)F	39718,07	-6,95583	62,12372	1068,60289	1,33197	52,21963	3,02733
Training	CHEMBL454233	CS(=O)(=O)Nc1ccc(SSc2ccc(NS(=O)(=O)C)cc2)cc1	100000	-8,59527	91,63683	1464,65998	1,47785	37,13336	2,10776
Training	CHEMBL1630812	O=C(COc1ccccc1)Nc2cccc(c2)C(=O)C(=O)c3ccccn3	100000	-9,6808	101,65147	1640,80899	0,95357	67,46817	0
Training	BUTANEDIONE	CC(=O)C(=O)C	100000	-4,12641	38,99191	477,56011	1,08943	13,52361	1,6302
Training	CHLORANIL	ClC1=C(Cl)C(=O)C(=C(Cl)C1=O)Cl	83,9	-6,01683	35,44918	775,60369	1,15462	21,84609	1,62998
Training	CHEMBL242930	Fc1cc(F)c(F)c(c1)C(=O)C(=O)c2cc(F)cc(F)c2F	140	-10,17763	78,88504	1394,74629	0,70475	66,29793	0,39676
Training	CHEMBL192410	Fc1ccc(cc1)C(=O)C(=O)c2ccc(F)cc2	230,03	-8,84043	62,47769	1155,77061	0,40767	67,53519	0,31049
Training	CHEMBL372716	O=C(C(=O)c1ccsc1)c2ccsc2	1046,03	-6,19953	55,30085	933,6043	1,83188	15,98135	2,51856
Training	CHEMBL235289	[O-][N+](=O)c1ccc2C(=O)C(=O)c3ccc(c1c23)[N+](=O)[O-]	570	-8,93688	75,20206	1257,80195	4,57696	36,96437	5,73787
Training	CHEMBL467450	CCCCCCCCCCCCS(=O)(=O)CC(O)(O)C(F)(F)F	370,85	-7,54174	89,72753	1446,83763	2,20111	80,1081	3,57785
Training	CHEMBL195713	Fc1c(F)c(F)c(C(=O)C(=O)c2c(Cl)c(Cl)c(Cl)c(Cl)c2Cl)c(F)c1F	1694,12	-9,61768	70,06538	1580,22519	0,93104	41,18243	0
Training	CHEMBL222292	Clc1ccc2C(=O)C(=O)Nc2c1Cl	9400	-7,27826	45,72829	839,11535	1,0877	28,63444	2,10311
Training	5-IODOISATIN	Ic1ccc2NC(=O)C(=O)c2c1	26900	-6,8182	41,31325	768,31659	0,91933	30,1539	1,87761
Training	CHEMBL521072	OC(=O)c1ccc(cc1)S(=O)(=O)Nc2ccc(NS(=O)(=O)c3ccc(F)cc3)cc2	100000	-10,17239	112,18676	1772,46981	1,71105	58,998	0,97427
Training	CHEMBL190496	[O-][S+](Cc1ccccc1)Cc2ccccc2	100000	-8,2962	56,03473	1044,45518	0,59009	71,43718	1,44023
Training	CHEMBL520371	CNC(=O)c1ccc(cc1)S(=O)(=O)Nc2ccc(NS(=O)(=O)c3ccc(F)cc3)cc2	100000	-10,68383	120,11853	1855,8735	1,80699	58,5081	1,16124
Training	CHEMBL484275	O=S(=O)(N(c1ccccc1)c2ccccc2)c3ccccc3	100000	-9,10446	75,01654	1356,08542	1,21192	73,72673	0
Training	CHEMBL487300	Brc1cccc(c1)S(=O)(=O)Nc2ccc3c(Cc4cc(NS(=O)(=O)c5cccc(Br)c5)ccc34)c2	100000	-12,10734	117,39148	2058,11207	2,52745	79,72865	1,51442
Training	CHEMBL3526990	COc1ccc2cc(ccc2c1)[C@@H](C)C(=O)O[C@@H]3O[C@@H]([C@@H](O)[C@H](O)[C@H]3O)C(=O)O	100000	-11,11173	109,79326	1866,90165	3,6457	72,24584	3,41673
Training	9-10-PHENANTHRENEQUINONE	O=C1C(=O)c2ccccc2c3ccccc13	2,5	-9,15798	49,37602	1042,36351	1,22699	61,8869	1,50656
Training	CHEMBL89506	CCCCCCCCSCC(=O)C(F)(F)F	97,36	-6,31853	64,69609	1057,20346	0,79974	62,87613	1,24182
Training	CHEMBL1812857	CCCCCCC(=O)C(=O)CCCCCC	4,8	-6,61702	63,40497	1053,08	1,23672	69,73581	3,02827
Training	CHEMBL1630808	Clc1ccc(C(=O)Nc2cccc(c2)C(=O)C(=O)c3ccccn3)c(Cl)c1	279	-10,1408	86,71536	1608,67069	1,20882	61,74949	0,98422
Training	CHEMBL223000	BrCCN1C(=O)C(=O)c2ccccc12	290	-7,18139	44,90503	925,69225	0,94279	34,05626	2,12802
Training	CHEMBL242287	Fc1ccc(C(=O)C(=O)c2ccc(F)cc2F)c(F)c1	370	-9,39971	69,98093	1282,51985	0,71934	64,06801	0,51639
Training	CHEMBL244427	OC(C(=O)c1cccc(F)c1F)c2cccc(F)c2F	730	-9,55225	74,75573	1273,89213	0,76589	69,68596	0,3413
Training	CHEMBL235931	O=C1C(=O)c2ccccc2CCc3ccccc13	2980	-9,72746	49,45916	1150,58412	1,27326	72,40147	0,95593
Training	CHEMBL373715	Cc1ccc(Cl)c2C(=O)C(=O)Nc12	5530	-7,12989	41,02356	832,87307	1,20922	32,52004	2,13491
Training	CHEMBL222988	Clc1cc2NC(=O)C(=O)c2cc1Cl	21000	-7,27292	52,69779	823,78257	1,16944	27,00356	2,01992
Training	CHEMBL244632	OC(C(=O)c1cccc(c1)C(F)(F)F)c2cccc(c2)C(F)(F)F	100000	-10,40927	85,06156	1524,67593	1,67918	100,85855	0,74713
Training	CHEMBL482853	Cc1ccc(cc1)S(=O)(=O)Nc2ccc(NS(=O)(=O)c3ccc(F)cc3)cc2	100000	-10,19811	88,35935	1676,16648	1,49263	69,03322	1,39827
Training	CHEMBL487304	Clc1ccc(cc1)S(=O)(=O)Nc2cc(Br)c(NS(=O)(=O)c3ccc(Cl)cc3)cc2Br	100000	-9,72666	108,67906	1741,83707	2,57374	63,69442	0,36681
Training	CHEMBL425192	Oc1ccc(cc1)C(=O)C(=O)c2ccccc2	100000	-8,4285	61,92325	1094,46674	0,77044	67,26749	0,60122
Training	CHEMBL363855	CC(=O)CCC(=O)c1ccccc1	100000	-7,04131	52,18567	820,22424	0,58465	62,99944	1,50499
Training	CHEMBL365129	O=S(=O)(Cc1ccccc1)Cc2ccccc2	100000	-8,31476	51,30865	1097,37322	0,56236	66,85456	1,54079
Training	CHEMBL365982	OC(C(O)c1ccccc1)c2ccccc2	100000	-8,31302	48,37341	1050,57532	1,26137	63,23178	2,50901
Training	CHEMBL375078	OC(=O)c1cccc2C(=O)C(=O)Nc12	100000	-7,25407	46,6217	929,79734	0,95215	23,43784	2,04596
Training	CHEMBL1812864	CCCCCCC(=O)C(=O)c1ccccc1	2,19	-7,25153	57,45385	1038,35983	1,57277	65,91577	3,1172
Training	CHEMBL1812858	CCCCCCCC(=O)C(=O)CCCCCCC	7,6	-6,92774	70,63703	1171,35875	1,3073	83,39094	3,08587
Training	CHEMBL376291	Clc1ccc(CN2C(=O)C(=O)c3ccccc23)cc1	25	-9,43743	57,22507	1211,6639	1,08892	60,42754	1,78742
Training	CHEMBL190672	Brc1csc(c1)C(=O)C(=O)c2cc(Br)cs2	20,8	-7,01682	55,14388	1043,17607	1,49911	22,21123	2,36969
Training	ACENAPHTHOQUINONE	O=C1C(=O)c2cccc3cccc1c23	31	-8,32898	47,02397	899,76133	1,266	55,02302	1,95589
Training	CHEMBL376518	ICCN1C(=O)C(=O)c2ccccc12	130	-7,23481	46,40918	921,60276	1,0198	35,26775	2,21368
Training	CHEMBL244631	OC(C(=O)c1ccc(F)c(F)c1F)c2ccc(F)c(F)c2F	260	-9,76963	69,09919	1407,20106	0,80633	63,5203	0,37085
Training	CRYPTOTANSHINONE	C[C@H]1COC2=C1C(=O)C(=O)c3c4CCCC(C)(C)c4ccc23	544	-10,21961	82,03402	1380,38453	4,15024	91,91619	0,7587
Training	CHEMBL190660	OC(C(=O)c1cccs1)c2cccs2	3100	-6,55369	49,90006	920,50715	0,82761	19,30528	2,04405
Training	CHEMBL376228	Clc1cccc2NC(=O)C(=O)c12	8250	-6,84895	44,84087	776,40801	1,0678	24,80412	2,18125
Training	CHEMBL224822	Clc1ccc2C(=O)C(=O)Nc2c1	53900	-7,11905	48,33732	767,04093	1,15702	25,01615	2,61782
Training	CHEMBL521499	Fc1ccc(cc1F)S(=O)(=O)Nc2ccc(NS(=O)(=O)c3ccc(F)c(F)c3)cc2	100000	-10,55277	95,05534	1789,97954	1,30968	60,91503	1,03593
Training	CHEMBL1812853	CCC(=O)C(=O)CC	100000	-4,72713	47,68965	550,90721	0,99669	21,03565	1,90564
Training	CHEMBL191625	CC(=O)CC(=O)C	100000	-4,45472	36,84827	473,58221	1,03204	16,25712	2,59201
Training	CHEMBL192712	CC1NC(=O)C(=O)OC1C	100000	-5,67985	35,84466	627,77361	1,24499	9,80967	3,17902
Training	CHEMBL483471	Fc1c(F)c(NS(=O)(=O)c2ccc(Cl)cc2)c(F)c(F)c1NS(=O)(=O)c3ccc(Cl)cc3	100000	-10,09832	111,86579	1985,96459	2,99734	55,30692	0
Training	CHEMBL483683	Clc1cccc(c1Cl)S(=O)(=O)Nc2ccc(NS(=O)(=O)c3cccc(Cl)c3Cl)cc2	100000	-10,24157	95,58368	1779,25541	1,59022	69,68574	0,26803
Training	CHEMBL489005	Fc1ccc(cc1)S(=O)(=O)Nc2c(F)c(F)c(NS(=O)(=O)c3ccc(F)cc3)c(F)c2F	100000	-10,29073	90,15174	1904,15529	3,32528	50,96264	3,19972
Training	CHEMBL489652	Cc1c(C)c(NS(=O)(=O)c2cccc(Cl)c2)c(C)c(C)c1NS(=O)(=O)c3cccc(Cl)c3	100000	-9,85933	93,31575	1766,38264	2,3339	81,14871	0
Training	CHEMBL448073	Clc1cc(NS(=O)(=O)c2ccccc2)ccc1NS(=O)(=O)c3ccccc3	100000	-9,61308	81,32666	1606,52109	0,94661	74,29744	0
Training	CHEMBL192474	COc1ccc(cc1)C(=O)C(=O)c2ccccc2	174,39	-8,3446	61,49001	1161,11987	0,45976	66,52489	0,29738
Training	CHEMBL373345	O=C(C(=O)c1ccccn1)c2ccccn2	245,3	-7,64191	53,37354	1036,94716	1,29264	37,13756	2,59224
Training	CHEMBL223039	Clc1ccc(Cl)c2C(=O)C(=O)Nc12	590	-6,92821	40,83831	830,20023	1,21315	25,88192	2,22316
Training	CHEMBL284028	Oc1nc2cc(Cl)c(Cl)cc2nc1O	100000	-7,86709	54,18135	899,41961	1,25211	27,31776	2,37687
Training	CHEMBL223254	Cc1ccc(C)c2C(=O)C(=O)Nc12	100000	-7,3083	41,73751	832,56208	1,26424	36,90917	2,20615
Training	CHEMBL192648	OC(=O)c1ccc(cc1)C(=O)c2ccccc2	100000	-8,29335	59,47996	1106,99839	1,03227	59,87416	1,17707
Training	CHEMBL485107	Fc1cc(cc(F)c1F)S(=O)(=O)Nc2ccc(NS(=O)(=O)c3cc(F)c(F)c(F)c3)cc2	100000	-10,86516	100,76826	1907,87498	1,85734	59,85524	1,2118
Training	CHEMBL485245	Fc1ccc(cc1F)S(=O)(=O)Nc2ccc(NS(=O)(=O)c3ccccc3)cc2	100000	-10,02849	90,18407	1672,51717	1,41778	62,53762	1,21192
Training	CHEMBL486072	Clc1cccc(c1)S(=O)(=O)Nc2ccc3c(Cc4cc(NS(=O)(=O)c5cccc(Cl)c5)ccc34)c2	100000	-12,04778	115,22662	2060,87248	2,39817	78,79318	1,34981
Training	CHEMBL363919	O=C1CCCC(=O)C1	100000	-5,37755	49,66396	591,21273	0,90854	25,30443	0,74657
Training	CHEMBL365517	[O-][N+](=O)c1ccc(C(=O)C(=O)c2ccccc2)c(c1)[N+](=O)[O-]	100000	-9,23217	75,42609	1411,92088	1,29317	65,04291	1,31512
Training	CHEMBL460807	CCCCCCCCS(=O)(=O)CC(O)(O)C(F)(F)F	757,08	-7,56352	75,38734	1241,63537	1,48272	60,5622	3,44483
Training	CHEMBL1812862	CCCCC(=O)C(=O)c1ccccc1	26,05	-6,8967	59,58244	876,53277	0,69806	74,46586	0,42885
Training	CHEMBL365161	CC(C)(C)C1=CC(=O)C(=O)C(=C1)C(C)(C)C	88,6	-7,54497	50,02609	992,82807	2,69404	53,20517	3,27401
Training	CHEMBL371858	Cc1ccc(cc1)C(=O)C(=O)c2ccccc2	125,44	-8,81266	58,44062	1108,59052	1,25533	64,8718	2,28817
Training	CHEMBL3527338	O[C@@H]1[C@@H](O)[C@H](OC(=O)Cc2ccccc2Nc3c(Cl)cccc3Cl)O[C@@H]([C@H]1O)C(=O)O	4320	-10,07536	93,06405	1775,11889	2,08235	61,23043	2,28477
Training	CHEMBL115897	Brc1ccc2NC(=O)C(=O)c2c1	31700	-6,89485	40,42945	769,94806	0,8799	28,01215	2,11354
Training	5-METHOXYISATIN	COc1ccc2NC(=O)C(=O)c2c1	100000	-6,86966	46,09745	831,25724	0,9931	18,23128	2,62085
Training	CHEMBL521292	COc1ccc(cc1)S(=O)(=O)Nc2ccc(NS(=O)(=O)c3ccc(F)cc3)cc2	100000	-9,91816	94,91378	1745,42485	1,7288	59,02893	1,67345
Training	CHEMBL509333	Cc1cc(NS(=O)(=O)c2ccccc2)ccc1NS(=O)(=O)c3ccccc3	100000	-9,69415	84,01557	1602,61738	1,59172	65,53519	1,51066
Training	CHEMBL482441	Clc1ccc(cc1Cl)S(=O)(=O)Nc2ccc(NS(=O)(=O)c3ccc(Cl)c(Cl)c3)cc2	100000	-10,30464	108,5474	1749,13992	1,87978	66,55374	0,48516
Training	CHEMBL483467	Cc1c(C)c(NS(=O)(=O)c2cccc(Br)c2)c(C)c(C)c1NS(=O)(=O)c3cccc(Br)c3	100000	-9,7264	95,3546	1761,55947	2,69783	83,5689	0
Training	CHEMBL491262	Fc1cc(cc(F)c1F)S(=O)(=O)Nc2ccc3c(Cc4cc(NS(=O)(=O)c5cc(F)c(F)c(F)c5)ccc34)c2	100000	-12,21651	96,64965	2052,28713	1,85053	91,54508	1,40622
Training	CHEMBL388883	FC(F)(F)c1ccc(c(c1)C(=O)C(=O)c2cc(ccc2C(F)(F)F)C(F)(F)F)C(F)(F)F	100000	-10,83295	88,54137	1906,52379	2,09557	87,91442	1,64382
Training	CHEMBL363186	CC1C(=O)CCC1=O	100000	-5,30804	51,58373	617,28834	1,36311	27,37367	0,80857
Training	CHEMBL460806	CCCCCCCC[S+]([O-])CC(O)(O)C(F)(F)F	7395,84	-7,1566	74,67889	1155,48559	1,68066	66,26914	3,08658
Training	CHEMBL86668	CCCCCCCCCCC(=O)C(F)(F)F	76,83	-6,5603	68,5352	1018,35278	1,09329	75,26608	1,68243
Training	CHEMBL1812863	CCCCCC(=O)C(=O)c1ccccc1	6,68	-7,02908	60,00363	952,92852	0,58574	76,59413	0,43411
Training	CHEMBL244633	Fc1ccccc1C(=O)C(=O)c2ccccc2F	185	-8,98943	65,00572	1156,0597	0,72896	73,83614	0,53536
Training	CHEMBL1630810	Cc1ccc(cc1)C(=O)Nc2cccc(c2)C(=O)C(=O)c3ccccn3	1034	-10,02729	84,5075	1536,96104	1,41236	65,64866	1,5539
Training	CHEMBL222190	Clc1cccc2C(=O)C(=O)Nc12	11400	-6,87177	41,37606	770,2878	1,14521	27,8583	2,40852
Training	CHEMBL242506	FC(F)(F)c1cc(cc(c1)C(F)(F)F)C(=O)C(=O)c2cc(cc(c2)C(F)(F)F)C(F)(F)F	100000	-11,59486	96,88157	1876,40598	0,75665	109,82981	0
Training	CHEMBL225175	OCN1C(=O)C(=O)c2ccccc12	100000	-7,04745	44,37841	840,14842	1,4046	31,62321	2,75691
Training	CHEMBL519501	Clc1ccc(Cl)c(c1)S(=O)(=O)Nc2ccc(NS(=O)(=O)c3cc(Cl)ccc3Cl)cc2	100000	-9,74867	88,30354	1740,04383	1,88613	64,66121	0,63227
Training	CHEMBL482854	Cc1ccc(cc1)S(=O)(=O)Nc2ccc(NS(=O)(=O)c3cc(F)ccc3F)cc2	100000	-10,41524	96,57598	1723,32989	1,68995	71,14526	1,15313
Training	CHEMBL433282	[O-][N+](=O)c1ccc2c(c1)C(=O)C(=O)c3ccccc23	15,9	-9,68904	57,75709	1232,92561	1,2573	57,59118	1,49805

Training	CHEMBL221512	CCCCCCCCCCCCCCCCN1C(=O)C(=O)c2ccccc12	All_Docked_Molecules 16	-8,01086	107,72733	1638,17746	1,74296	111,43841	0,53431	
Training	CHEMBL222712	Clc1ccc(NCN2C(=O)C(=O)c3ccccc23)cc1	610	-9,06915	68,41631	1267,98978	2,57981	65,45268	2,66608	
Training	CHEMBL1812852	BrC(C(=O)C(=O)Cc1ccccc1)c2ccccc2	2630	-9,11049	71,18444	1210,43703	1,39181	87,49844	1,87402	
Training	CHEMBL1812850	O=C(Cc1ccccc1)C(=O)Cc2ccccc2	3080	-9,01239	69,32227	1158,5353	1,01023	82,83272	1,95684	
Training	CHEMBL396977	OC(C(=O)c1cccc(F)c1)c2cccc(F)c2	3130	-8,87956	57,89638	1146,65825	0,57402	67,2408	1,00359	
Training	TANSHINONEIIA	Cc1coc2c3ccc4c(CCCC4(C)C)c3C(=O)C(=O)c12	6890	-10,65115	83,03919	1390,84181	3,78031	94,53799	0,65616	
Training	CHEMBL222999	ClN1C(=O)C(=O)c2ccccc12	22800	-7,02208	38,97218	794,36285	0,97635	31,00567	2,16367	
Training	CHEMBL376290	Cc1cc(Cl)cc2C(=O)C(=O)Nc12	34100	-7,28986	44,74798	834,10496	1,14101	40,14914	1,60746	
Training	CHEMBL222849	O=C1N(CN2CCOCC2)c3ccccc3C1=O	100000	-8,25688	53,72957	1180,0811	1,19354	34,44219	1,73797	
Training	CHEMBL190613	CC1CCCC(=O)C1=O	100000	-5,59867	37,48361	579,28499	1,29202	30,6896	2,19439	
Training	CHEMBL191739	[O-][N+](=O)c1cc(ccc1Br)C(=O)C(=O)c2ccc(Br)c(c2)[N+](=O)[O-]	100000	-8,80545	74,92942	1398,40239	2,35987	39,73345	3,55997	
Training	CHEMBL482640	Clc1cccc(c1)S(=O)(=O)Nc2ccc(NS(=O)(=O)c3cccc(Cl)c3)cc2	100000	-9,70491	80,87825	1652,39388	2,06907	47,68055	3,03537	
Training	CHEMBL483470	Clc1c(Cl)c(NS(=O)(=O)c2ccccc2)c(Cl)c(Cl)c1NS(=O)(=O)c3ccccc3	100000	-9,39526	79,67202	1655,08703	1,1028	64,15714	0	
Training	CHEMBL489006	Fc1c(F)c(NS(=O)(=O)c2ccc(Br)cc2)c(F)c(F)c1NS(=O)(=O)c3ccc(Br)cc3	100000	-9,83242	114,53235	1976,72148	3,52506	56,3565	0	
Training	CHEMBL491256	O=S(=O)(Nc1ccc(NS(=O)(=O)c2ccc3ccccc3c2)cc1)c4ccc5ccccc5c4	100000	-12,4223	113,4273	1948,04627	2,27129	107,64897	1,42671	
Training	CHEMBL395652	Cc1cc(C)c(C(=O)C(=O)c2c(C)cc(C)cc2C)c(C)c1	100000	-9,73159	69,11409	1264,66116	0,93945	85,85233	0,39799	
Training	DIHYDROTANSHINONEI	C[C@H]1COC2=C1C(=O)C(=O)c3c2ccc4c(C)cccc34	398	-10,87049	79,48147	1328,96748	2,92162	84,59782	1,16527	
Training	CHEMBL1812854	CCCC(=O)C(=O)CCC	8060	-5,11953	42,07517	658,15358	0,67649	33,76783	1,89062	
Training	CHEMBL364588	CCCCC(=O)C(=O)C	16900	-5,21158	41,83676	588,52844	0,71616	35,71157	2,14004	
Training	TANSHINONEI	Cc1coc2c3ccc4c(C)cccc4c3C(=O)C(=O)c12	26250	-11,53381	75,47291	1329,89692	1,99872	88,7735	0,9555	
Training	CHEMBL223001	CC(=O)N1C(=O)C(=O)c2ccccc12	36000	-7,50212	36,92989	897,25844	0,78	30,44188	1,95723	
Training	CHEMBL224988	Fc1cccc2C(=O)C(=O)Nc12	100000	-6,92735	43,62028	764,66742	1,21507	24,17892	2,73677	
Training	DIPHENYLETHANEDIOAT	O=C(Oc1ccccc1)C(=O)Oc2ccccc2	100000	-8,26606	62,35021	1170,24232	1,42793	60,23564	2,5803	
Training	CHEMBL483072	Cc1ccc(c(F)c1)S(=O)(=O)Nc2ccc(NS(=O)(=O)c3ccc(C)cc3F)cc2	100000	-10,79531	96,60327	1773,72953	1,65207	76,22325	1,22252	
Training	CHEMBL483468	Cc1c(C)c(NS(=O)(=O)c2ccc(Br)cc2)c(C)c(C)c1NS(=O)(=O)c3ccc(Br)cc3	100000	-9,88934	96,84396	1867,00686	2,93811	75,28461	0,19153	
Training	CHEMBL484698	Fc1cccc(F)c1S(=O)(=O)Nc2ccc(NS(=O)(=O)c3c(F)cccc3F)cc2	100000	-10,1318	87,3823	1756,28232	1,1867	61,10077	0,63553	
Training	CHEMBL485106	Fc1cc(F)cc(c1)S(=O)(=O)Nc2ccc(NS(=O)(=O)c3cc(F)cc(F)c3)cc2	100000	-10,41721	95,69187	1780,82797	1,21241	68,57249	0,16486	
Training	CHEMBL487299	O=S(=O)(Nc1ccc2c(Cc3cc(NS(=O)(=O)c4ccccc4)ccc23)c1)c5ccccc5	100000	-11,5145	115,52027	1931,30379	3,96952	86,63904	3,04228	
Training	CHEMBL222644	CCCCCCCCCCCCN1C(=O)C(=O)c2ccccc12	10	-8,10441	90,66324	1402,18485	0,45441	92,48233	0	
Training	CHEMBL225169	CC(=C)CN1C(=O)C(=O)c2cc(Br)ccc12	66	-7,88211	50,52575	1040,2673	1,11783	45,96303	1,65776	
Training	CHEMBL437373	OC(C(=O)c1cc(F)cc(F)c1)c2cc(F)cc(F)c2	170	-9,33219	66,65619	1266,183	0,96456	64,63266	1,04354	
Training	CHEMBL242768	OC(C(=O)c1ccccc1F)c2ccccc2F	990	-8,86615	62,22238	1156,58202	0,60485	72,59447	0,35214	
Training	CHEMBL374331	Cc1cc(C)c(NCN2C(=O)C(=O)c3ccccc23)cc1C	1620	-10,30575	75,11938	1404,29103	2,93895	85,56504	2,83148	
Training	CHEMBL374970	CCCC(=O)N1C(=O)C(=O)c2ccccc12	7690	-8,09159	47,64461	1058,84915	1,04872	40,89518	2,26171	
Training	CHEMBL223785	COc1cccc2C(=O)C(=O)Nc12	100000	-6,70779	45,07508	832,68627	1,04356	21,58913	2,24624	
Training	CHEMBL236337	CCOc1ccc(cc1)C(=O)C(=O)c2ccc(OCC)cc2	100000	-8,3275	83,55114	1415,47811	1,36635	70,10101	0,26222	
Training	CHEMBL222795	O=C1N(CN2CCSCC2)c3ccccc3C1=O	100000	-7,94447	60,57893	1177,74461	1,42935	34,23073	1,10103	
Training	CHEMBL519326	Cc1ccc(C)c(c1)S(=O)(=O)Nc2ccc(NS(=O)(=O)c3ccc(F)cc3)cc2	100000	-10,58183	89,58947	1726,33829	1,09882	81,8099	0,43704	
Training	CHEMBL482639	Fc1ccc(cc1)S(=O)(=O)Nc2ccc(NS(=O)(=O)c3ccc(F)cc3)cc2	100000	-9,91398	84,37457	1675,74195	1,42618	62,59533	1,2816	
Training	CHEMBL487303	Brc1cc(NS(=O)(=O)c2ccccc2)c(Br)cc1NS(=O)(=O)c3ccccc3	100000	-9,37614	109,39549	1613,82843	2,74307	68,75286	0,59196	
Training	5-FLUOROISATIN	Fc1ccc2NC(=O)C(=O)c2c1	100000	-6,98971	42,62993	764,99992	1,06004	23,27442	2,72462	
Training	CHEMBL451609	Brc1ccc(cc1)S(=O)(=O)Nc2ccc(Oc3ccccc3)cc2	100000	-9,48624	94,88829	1540,82115	3,94211	79,51929	2,48209	
Training	VALPROICACID	CCCC(CCC)C(=O)O	100000	-5,44203	47,6216	634,47801	0,8894	41,21551	2,33102	
Training	CHEMBL1812861	CCCC(=O)C(=O)c1ccccc1	102	-6,85861	57,39825	823,73328	0,39508	65,66831	0,337	
Training	CHEMBL244426	OC(C(=O)c1c(F)cccc1F)c2c(F)cccc2F	190	-9,17971	64,59372	1264,72275	0,6951	64,59867	0,49404	
Training	CHEMBL242722	Fc1cc(F)c(F)c(c1)C(=O)C(=O)c2c(F)ccc(F)c2F	320	-10,05829	79,716	1390,05631	0,83413	64,61354	0,43616	
Training	CHEMBL1629724	FC(F)(F)c1ccc(cc1)C(=O)Nc2cccc(c2)C(=O)C(=O)c3ccccn3	437	-10,50789	113,47581	1768,30686	2,21399	79,35597	0,209	
Training	1-2-NAPHTHOQUINONE	O=C1C=Cc2ccccc2C1=O	930	-7,38528	40,54525	790,70736	1,04784	38,06613	2,40279	
Training	CHEMBL254426	Clc1ccc(cc1)S(=O)(=O)Nc2ccc(NS(=O)(=O)c3ccc(Cl)cc3)cc2	13700	-9,97087	90,35637	1676,34846	1,88587	66,59961	1,45941	
Training	CHEMBL222891	ClCC(=O)N1C(=O)C(=O)c2ccccc12	100000	-7,82257	45,24851	990,62984	1,02852	32,11835	2,21126	
Training	CHEMBL191676	CN(C)c1ccc(cc1)C(=O)C(=O)c2ccc(cc2)N(C)C	100000	-8,44461	70,06946	1367,68077	0,40859	48,81685	0	
Training	CHEMBL191688	CC1C(=O)CCCC1=O	100000	-5,50388	56,65683	658,46768	1,65693	32,30165	0,5927	
Training	CHEMBL482650	Cc1ccc(cc1)S(=O)(=O)Nc2ccc(NS(=O)(=O)c3ccc(Cl)cc3)cc2	100000	-10,19602	91,59329	1677,99631	1,87271	73,24021	1,46336	
Training	CHEMBL366205	Oc1ccc(Br)cc1C(=O)C(=O)c2cc(Br)ccc2O	100000	-8,82981	78,37249	1283,23772	2,50857	64,29595	1,40238	
Testing	CHEMBL1812856	CCCCCC(=O)C(=O)CCCCC	3	-6,24754	54,93243	932,99514	1,26851	56,55105	3,15328	
Testing	CHEMBL192592	Brc1ccc(s1)C(=O)C(=O)c2ccc(Br)s2	141,98	-7,61884	57,17362	1037,23339	1,43129	41,4575	2,24494	
Testing	PHENYLISATIN	O=C1N(c2ccccc2)c3ccccc3C1=O	23	-8,86891	53,75642	1089,19159	1,13951	57,13068	1,38907	
Testing	CHEMBL192241	BrCc1ccc(cc1)C(=O)C(=O)c2ccccc2	77,61	-8,74686	64,50339	1160,72811	0,46624	79,55058	0,19784	
Testing	CHEMBL482848	Brc1ccc(cc1)S(=O)(=O)Nc2ccc(NS(=O)(=O)c3ccc(Br)cc3)cc2	100000	-9,90877	95,82121	1664,5705	2,32445	68,29336	1,61678	
Testing	CHEMBL1630809	O=C(Cc1cccs1)Nc2cccc(c2)C(=O)C(=O)c3ccccn3	100000	-9,2162	94,90246	1587,44173	1,59586	57,52136	0,35206	
Testing	CHEMBL374905	O=C1N(CN2CCCCC2)c3ccccc3C1=O	100000	-8,38563	57,60709	1165,54336	1,89222	52,27821	1,81642	
Testing	CHEMBL460809	FC(F)(F)C(=O)CSCCc1ccccc1	63,34	-7,56511	68,97841	1000,86897	1,2024	69,04085	2,03073	
Testing	CHEMBL394806	OC(C(=O)c1cc(F)c(F)c(F)c1)c2cc(F)c(F)c(F)c2	1000	-9,50351	74,85337	1392,42779	1,07413	63,55685	0	
Testing	CHEMBL386000	CCC(=O)N1C(=O)C(=O)c2ccccc12	17500	-7,92868	44,65778	991,68773	1,03556	35,91073	2,21782	
Testing	5-NITROISATIN	[O-][N+](=O)c1ccc2NC(=O)C(=O)c2c1	100000	-7,10751	54,20239	865,31736	1,33275	17,46395	2,79226	
Testing	CHEMBL333636	Cc1cc(C)c2NC(=O)C(=O)c2c1	100000	-7,57443	44,87819	832,99117	1,03149	50,6784	1,30957	
Testing	CHEMBL395018	Fc1cccc(C(=O)C(=O)c2cccc(F)c2F)c1F	34,7	-9,58637	74,24844	1276,44123	0,7217	70,56044	0,30052	
Testing	CHEMBL192625	Cc1ccc(cc1)C(=O)C(=O)c2ccc(C)cc2	534,51	-9,1261	65,37626	1163,14344	1,56545	72,40192	2,00994	
Testing	CHEMBL449475	CCCCCCOCC(O)(O)C(F)(F)F	1656,15	-6,53216	65,51628	942,9431	1,91354	44,40538	4,15964	
Testing	CHEMBL192039	Oc1ccc(C(=O)C(=O)c2ccc(O)cc2O)c(O)c1	100000	-9,13498	72,68463	1289,60082	1,56429	48,32886	1,90619	
Testing	CHEMBL483676	Fc1cccc(c1)S(=O)(=O)Nc2ccc(NS(=O)(=O)c3cccc(F)c3)cc2	100000	-10,03936	88,50406	1650,30413	0,83819	70,25276	0,2433	
Testing	CHEMBL487102	Clc1ccc(cc1)S(=O)(=O)Nc2cc(Cl)c(NS(=O)(=O)c3ccc(Cl)cc3)cc2Cl	100000	-10,11857	106,87188	1746,23654	1,99488	65,08216	0,44487	
Testing	CHEMBL3526740	CC(C)Cc1ccc(cc1)C(C)C(=O)O[C@@H]2O[C@@H]([C@@H](O)[C@H](O)[C@H]2O)C(=O)O	100000	-10,22894	90,95085	1740,92489	2,15897	76,50062	2,26227	
Testing	CHEMBL375304	Cc1cccc(NC(=O)CN2C(=O)C(=O)c3ccccc23)c1	4350	-9,85315	79,52682	1371,89154	2,33123	70,77592	1,95778	
Testing	CHEMBL464002	CCCCCCCCCC[S+]([O-])CC(O)(O)C(F)(F)F	2624,29	-7,09813	79,52837	1281,61814	1,77009	68,5998	2,93414	
Testing	CHEMBL521464	Cc1c(C)c(NS(=O)(=O)c2ccc(Cl)cc2)c(C)c(C)c1NS(=O)(=O)c3ccc(Cl)cc3	100000	-9,96525	87,38081	1764,93468	1,12984	62,71265	0	
Testing	CHEMBL1630814	Clc1cccc(c1)C(=O)Nc2cccc(c2)C(=O)C(=O)c3ccccn3	756	-9,9743	90,50977	1488,3113	1,60084	67,36463	1,67103	
Testing	CHEMBL221362	COc1cc(NCN2C(=O)C(=O)c3ccccc23)cc(OC)c1OC	19500	-8,16971	85,58595	1364,49914	2,04224	39,97034	2,16998	
Testing	CHEMBL244213	OC(C(=O)c1ccc(F)cc1)c2ccc(F)cc2	100000	-8,78031	61,83519	1154,32352	0,41529	66,76497	0,29853	
Testing	CHEMBL483071	Cc1ccc(cc1)S(=O)(=O)Nc2ccc(NS(=O)(=O)c3ccc(F)c(F)c3)cc2	100000	-10,60435	96,77738	1735,27728	1,51603	68,54294	1,36503	
Testing	CHEMBL364729	ClC1=C(Cl)C(=O)C(=O)C(=C1Cl)Cl	5440	-6,26821	34,45974	786,0034	1,67098	19,35056	2,9262	
Testing	CHEMBL242296	FC(F)(F)c1ccc(C(=O)C(=O)c2ccc(cc2C(F)(F)F)C(F)(F)F)c(c1)C(F)(F)F	100000	-11,21145	118,95273	2041,50921	5,39444	124,70009	2,03425	
Testing	BENZOQUINONE	O=C1C=CC(=O)C=C1	100000	-5,21018	51,21179	613,14007	1,37858	25,71883	0,81364	
Testing	2-3-PENTANEDIONE	CCC(=O)C(=O)C	100000	-4,42824	45,61054	524,90874	1,1001	18,60129	1,51489	
Testing	CHEMBL235503	O=C1C(=O)c2c3ccccc3cc4cccc1c24	1,5	-10,05359	59,06039	1147,36261	2,15335	83,49752	1,56274	
Testing	CHEMBL192589	[O-][N+](=O)c1ccc(cc1)C(=O)C(=O)c2ccccc2	214,4	-8,7589	64,94537	1237,94512	0,64678	68,75892	0,4182	
Testing	CHEMBL221873	Oc1ccc(NC(=O)CN2C(=O)C(=O)c3ccccc23)cc1	100000	-9,32726	67,18841	1372,89632	1,46936	42,43066	2,10984	
Testing	CHEMBL395019	FC(F)(F)c1cccc(c1)C(=O)C(=O)c2cccc(c2)C(F)(F)F	100000	-10,71262	85,20482	1534,60551	1,22956	100,69812	0,68403	
Testing	CHEMBL375126	Clc1ccc(CN2C(=O)C(=O)c3ccccc23)cc1Cl	31	-9,41782	62,68603	1269,50047	1,59854	57,37393	1,82488	
Testing	CHEMBL191822	COc1ccc(cc1OC)C(=O)C(=O)c2ccccc2Cl	3305,65	-8,48102	75,42	1304,02275	1,48446	46,4029	1,9991	
Testing	BENZOPHENONE	O=C(c1ccccc1)c2ccccc2	100000	-7,49358	61,0192	862,62293	1,08998	76,06098	0	
Testing	ISATIN	O=C1Nc2ccccc2C1=O	100000	-6,69973	40,53644	709,4325	0,98372	24,82974	2,65272	
Testing	CHEMBL242288	Fc1cccc(F)c1C(=O)C(=O)c2c(F)cccc2F	5650	-9,36868	71,69203	1268,58446	0,89033	67,4194	0,50901	
Testing	CHEMBL79466	NC(=O)CN1C(=O)C(=O)c2ccccc12	100000	-7,60129	54,33053	950,62637	0,66115	38,29703	1,47835	
Testing	CHEMBL1812859	CCCCCCCCC(=O)C(=O)CCCCCCCC	0,84	-7,09482	78,0361	1306,33163	1,64472	93,91368	3,22118	
Testing	CHEMBL270374	CCCCCCCCCCCSCC(=O)C(F)(F)F	66,4	-6,51651	80,24754	1135,21193	1,31263	71,59189	1,65336	
Testing	CHEMBL440542	CCCCCCSCC(=O)C(F)(F)F	168,19	-6,17636	61,04425	871,21518	0,84875	55,67523	1,97685	
Testing	CHEMBL1630816	Fc1ccc(cc1)C(=O)Nc2cccc(c2)C(=O)C(=O)c3ccccn3	381	-10,30669	91,17194	1621,52728	1,93808	73,0464	1,32567	
Testing	CHEMBL189727	O=C1CCCCC1=O	100000	-5,17226	52,15895	594,72185	1,86977	26,2988	1,52556	
Testing	CHEMBL192595	CN(C)c1ccc(cc1)C(O)C(=O)c2ccccc2	100000	-8,65683	70,60019	1226,78007	0,83373	69,40243	0,19274	
Testing	CHEMBL492066	Cc1c(C)c(NS(=O)(=O)c2ccc(Cl)c(Cl)c2)c(C)c(C)c1NS(=O)(=O)c3ccc(Cl)c(Cl)c3	100000	-10,13264	93,13675	1877,69628	1,78846	62,5716	0	
Testing	CHEMBL327012	Clc1ccc2NC(=O)C(=O)c2c1	1220	-6,88847	40,10683	768,52451	0,83805	25,80688	2,21161	
Testing	CHEMBL394557	OC(C(=O)c1ccc(F)c(F)c1)c2ccc(F)c(F)c2	1300	-9,28366	67,02124	1281,36263	0,49101	61,33547	0,31092	
Testing	CHEMBL118883	Cc1ccc2NC(=O)C(=O)c2c1	100000	-7,2336	41,77745	769,10526	0,98043	33,34978	2,44391	
Testing	CHEMBL394807	OC(C(=O)c1ccc(cc1)C(F)(F)F)c2ccc(cc2)C(F)(F)F	870	-10,00121	77,03005	1514,72002	2,08149	91,46699	1,55414	
Testing	CHEMBL1162998	Cc1ccc(cc1)S(=O)(=O)Nc2ccc(NS(=O)(=O)c3ccc(F)cc3F)cc2	100000	-10,36689	88,19685	1739,56221	1,48786	65,78758	1,43404	
Testing	CHEMBL222711	O=C1N(CNc2ccc3ccccc3c2)c4ccccc4C1=O	100000	-10,78964	85,88862	1497,33614	3,33134	97,24167	2,20835	
Testing	CHEMBL242932	FC(F)(F)c1ccc(cc1)C(=O)C(=O)c2ccc(cc2)C(F)(F)F	170	-10,00668	92,72456	1522,651	2,87678	92,4474	1,62129	
Testing	CHEMBL222851	Cc1ccc(NCN2C(=O)C(=O)c3ccccc23)cc1	2650	-9,44182	68,58307	1268,74443	2,50232	74,55132	2,73108	
Testing	CHEMBL374740	Clc1cc(Cl)c2NC(=O)C(=O)c2c1	3520	-7,03274	44,66872	833,24666	1,16722	33,48858	1,62141	
Testing	CHEMBL221830	Cc1cc2C(=O)C(=O)Nc2cc1Br	16500	-7,46587	53,4275	820,32119	1,17483	35,31358	1,84551	
Testing	CHEMBL1630813	COc1ccc(cc1)C(=O)Nc2cccc(c2)C(=O)C(=O)c3ccccn3	100000	-9,83324	92,41665	1650,60565	1,63576	62,9784	1,11124	
Testing	CHEMBL222752	O=C1N(Cc2ccc(Cc3ccc(CN4C(=O)C(=O)c5ccccc45)cc3)cc2)c6ccccc6C1=O	8	-12,48603	120,70946	1953,4562	3,82043	94,98092	4,05079	
Testing	CHEMBL492067	Cc1c(C)c(NS(=O)(=O)c2cc(Cl)cc(Cl)c2)c(C)c(C)c1NS(=O)(=O)c3cc(Cl)cc(Cl)c3	100000	-8,61935	116,184	1598,00583	3,7359	68,97554	0	
Testing	CHEMBL398109	O=C1C(=O)c2sccc2c3ccsc13	150	-6,81721	38,53684	883,18906	1,31648	14,19688	2,54539	
Testing	CHEMBL1630811	Fc1ccc(cc1F)C(=O)Nc2cccc(c2)C(=O)C(=O)c3ccccn3	1244	-10,39692	95,02829	1663,92453	2,29271	73,77421	1,38811	
Testing	CHEMBL235290	O=C1N2CCCc3cccc(C(=O)C1=O)c23	5380	-8,34246	58,26606	1027,40867	2,67368	43,45508	2,89061	
Testing	CHEMBL235504	O=C1C(=O)c2cccnc2c3ncccc13	5400	-8,20617	51,01352	1017,99211	1,12047	23,26617	2,16247	
	**In blue the ligands predicted as active in the ten X-ray structures**									
	**In red the ligand badly predicted as inactive in the ten X-ray structures**									

Table S2: List of drugs predicted as active with the MLP model									
ligand ID	Basename	Score	Gauss1	Gauss2	Repulsion	Hydrophobic	Hydrogen	GenericName	MLP_prediction
260	DB00425	-10,51	77,16	1391,39	0,66	67,56	0,01	Zolpidem	0,015209675
212	DB00366	-10,09	71,07	1204,98	0,54	68,17	0,00	Doxylamine	0,018482715
531	DB00758	-10,30	75,64	1255,61	0,65	60,32	0,14	Clopidogrel	0,018669128
439	DB00645	-10,39	74,46	1297,43	0,72	63,41	0,07	Dyclonine	0,019448757
259	DB00424	-9,85	75,04	1276,02	0,80	60,05	0,51	Hyoscyamine	0,020001113
641	DB00889	-10,68	74,85	1405,22	0,78	56,74	0,12	Granisetron	0,020923913
379	DB00572	-10,14	73,97	1302,85	0,88	60,97	0,56	Atropine	0,02151668
521	DB00748	-9,80	71,37	1220,44	0,70	60,30	0,03	Carbinoxamine	0,021750808
5554	DB08800	-10,21	67,33	1215,97	0,52	56,44	0,00	Chloropyramine	0,021885276
530	DB00757	-10,47	80,59	1477,67	0,75	57,30	0,75	Dolasetron	0,022793025
1005	DB01382	-10,28	71,30	1301,28	0,50	37,50	0,22	Glycodiazine	0,023188293
407	DB00608	-10,77	81,83	1340,52	0,68	61,19	0,00	Chloroquine	0,023351759
715	DB00979	-9,80	68,90	1285,82	0,80	61,58	0,65	Cyclopentolate	0,02349639
905	DB01205	-9,90	75,50	1340,64	0,61	34,61	0,31	Flumazenil	0,023601592
68	DB00196	-10,30	77,19	1373,05	0,81	47,32	0,17	Fluconazole	0,023849726
264	DB00429	-10,75	91,77	1612,03	3,96	67,15	0,94	Carboprost Tromethamine	0,023890376
183	DB00334	-10,45	72,38	1328,49	0,56	42,62	0,00	Olanzapine	0,024311751
728	DB00993	-8,62	63,02	1194,93	0,95	0,00	1,74	Azathioprine	0,024837106
181	DB00332	-10,89	81,85	1477,30	0,83	62,75	0,22	Ipratropium bromide	0,024902225
70	DB00198	-10,11	70,77	1318,56	0,84	49,73	0,59	Oseltamivir	0,02493745
444	DB00651	-8,31	67,16	1147,17	1,03	0,00	1,48	Dyphylline	0,025066495
561	DB00792	-9,98	68,13	1146,72	0,52	59,31	0,00	Tripelennamine	0,025817573
791	DB01069	-10,22	73,01	1195,53	0,57	55,12	0,00	Promethazine	0,027144492
739	DB01006	-10,75	81,64	1321,02	0,71	64,56	0,00	Letrozole	0,027419925
520	DB00747	-9,92	75,06	1341,11	0,86	45,02	0,59	Scopolamine	0,028185934
1152	DB01618	-10,27	72,05	1210,32	0,61	52,79	0,23	Molindone	0,02865082
793	DB01071	-10,93	71,67	1386,02	0,83	73,21	0,00	Mequitazine	0,028692693
434	DB00640	-8,65	72,40	1165,19	1,08	0,00	1,65	Adenosine	0,028808236
767	DB01042	-9,54	68,03	1158,22	0,61	37,67	0,58	Melphalan	0,028875798
283	DB00454	-9,72	69,66	1073,14	0,47	59,01	0,06	Meperidine	0,028987676
668	DB00922	-10,45	74,27	1293,60	0,64	46,85	0,05	Levosimendan	0,029135823
466	DB00679	-11,28	82,14	1477,00	0,70	56,79	0,00	Thioridazine	0,029509753
457	DB00669	-9,94	75,11	1248,88	0,56	34,12	0,17	Sumatriptan	0,029818773
102	DB00238	-9,87	67,16	1224,87	0,68	42,93	0,39	Nevirapine	0,029990375
67	DB00193	-10,01	70,12	1157,37	0,62	57,57	0,08	Tramadol	0,030144274
701	DB00963	-10,32	74,04	1238,44	0,85	63,85	0,64	Bromfenac	0,030560791
105	DB00242	-8,66	65,09	1174,21	0,87	8,36	1,25	Cladribine	0,030963808
3888	DB06766	-10,85	78,43	1372,28	0,87	65,40	0,09	Alcaftadine	0,030984342
1124	DB01580	-9,88	66,60	1180,42	0,76	45,50	0,79	Oxprenolol	0,03125897
302	DB00477	-10,41	76,07	1252,55	0,65	50,90	0,00	Chlorpromazine	0,031416625
1150	DB01615	-10,54	73,88	1386,17	0,92	53,04	0,50	Aceprometazine	0,031738847
760	DB01032	-9,41	66,92	1188,49	0,76	48,22	0,67	Probenecid	0,03186956
524	DB00751	-9,86	68,28	1138,79	0,59	73,86	0,08	Epinastine	0,031937778
219	DB00374	-10,94	96,55	1611,53	2,93	76,42	0,56	Treprostinil	0,032362312
735	DB01002	-9,71	66,57	1275,88	1,01	61,55	0,04	Levobupivacaine	0,03265944
151	DB00297	-9,71	66,57	1275,88	1,01	61,55	0,04	Bupivacaine	0,03265944
299	DB00474	-9,45	69,68	1190,40	0,70	51,40	0,29	Methohexital	0,03279066
834	DB01124	-9,73	68,95	1127,65	0,70	52,50	0,36	Tolbutamide	0,032902956
504	DB00724	-9,51	67,38	1079,78	0,59	53,31	0,29	Imiquimod	0,033648282
706	DB00969	-10,80	71,53	1341,96	0,63	49,08	0,24	Alosetron	0,03383127
5556	DB08802	-9,50	64,18	1237,98	0,59	38,08	0,00	Isothipendyl	0,034156322
255	DB00420	-10,19	74,58	1191,96	0,68	55,98	0,00	Promazine	0,034639418
653	DB00904	-10,76	66,79	1346,77	0,69	52,57	0,31	Ondansetron	0,035060912
341	DB00527	-11,48	79,96	1529,46	0,67	55,06	0,09	Dibucaine	0,0351758
970	DB01297	-9,31	70,72	1186,03	0,68	37,43	0,81	Practolol	0,035223037
693	DB00953	-10,17	78,67	1195,07	0,68	49,68	0,07	Rizatriptan	0,035530716
384	DB00579	-10,28	76,18	1200,26	0,76	73,02	0,11	Mazindol	0,036393672
932	DB01237	-10,23	73,20	1191,04	0,72	75,19	0,06	Bromodiphenhydramine	0,036459267
717	DB00981	-9,90	73,57	1248,31	0,67	37,88	0,14	Physostigmine	0,036919296
108	DB00247	-11,13	82,44	1555,93	1,04	57,63	0,18	Methysergide	0,037426144
125	DB00268	-9,55	71,25	1133,11	0,75	62,91	0,27	Ropinirole	0,03757152
3822	DB04843	-11,05	81,33	1451,37	1,61	70,29	0,13	Mepenzolate	0,037658036
1000	DB01366	-9,92	74,86	1299,92	0,99	44,89	0,67	Procaterol	0,037944257
280	DB00449	-11,44	87,87	1509,80	1,22	64,56	1,06	Dipivefrin	0,037974328
544	DB00772	-8,71	66,47	1182,90	0,66	11,32	0,69	Malathion	0,03843364
699	DB00961	-9,58	68,90	1075,00	0,57	58,51	0,02	Mepivacaine	0,038762033

3818	DB04838	-10,34	70,08	1220,17	0,90	69,04	0,73	Cyclandelate	0,039043576
197	DB00349	-10,55	76,01	1258,98	0,85	70,10	0,39	Clobazam	0,039065927
3868	DB06710	-9,85	63,66	1282,20	1,65	63,59	0,23	Methyltestosterone	0,03913513
1115	DB01559	-10,53	67,83	1276,34	0,80	57,89	0,30	Clotiazepam	0,039443254
558	DB00788	-9,66	64,69	1066,31	0,67	60,98	0,48	Naproxen	0,0396339
688	DB00946	-10,56	65,64	1285,15	1,01	68,04	0,11	Phenprocoumon	0,040037602
121	DB00264	-9,57	67,95	1179,62	0,71	43,60	0,24	Metoprolol	0,040742368
426	DB00631	-9,19	74,69	1207,37	0,88	8,05	1,31	Clofarabine	0,040746182
184	DB00335	-9,50	74,06	1168,52	0,82	45,29	0,84	Atenolol	0,041326433
698	DB00960	-9,52	70,71	1126,64	0,78	40,68	0,72	Pindolol	0,041538864
378	DB00571	-9,58	72,71	1181,47	0,78	69,24	0,42	Propranolol	0,042477638
664	DB00918	-10,73	80,06	1418,26	0,81	40,87	0,22	Almotriptan	0,042690963
601	DB00837	-11,10	81,25	1408,19	0,90	53,60	0,81	Progabide	0,04283285
327	DB00508	-11,44	82,86	1461,89	0,73	63,86	0,00	Triflupromazine	0,0428344
806	DB01087	-9,30	74,61	1145,96	0,66	47,65	0,28	Primaquine	0,042994022
636	DB00880	-8,44	63,90	1089,14	0,88	14,17	1,36	Chlorothiazide	0,0432384
572	DB00806	-9,55	70,18	1254,69	0,70	20,62	0,76	Pentoxifylline	0,04344067
423	DB00628	-11,01	69,58	1357,78	1,14	68,32	1,25	Clorazepate	0,043861
98	DB00234	-11,01	74,84	1385,70	0,97	72,49	0,19	Reboxetine	0,0440664
874	DB01171	-9,21	64,74	1108,89	0,60	42,38	0,36	Moclobemide	0,044588774
744	DB01011	-9,55	71,30	1032,28	0,55	57,03	0,26	Metyrapone	0,04467991
5562	DB08808	-9,78	68,50	1104,60	0,81	53,74	0,75	Bupranolol	0,044706393
732	DB00999	-8,45	63,57	1093,27	0,95	13,55	1,48	Hydrochlorothiazide	0,04485762
591	DB00827	-9,27	68,02	1212,28	0,86	19,07	1,12	Cinoxacin	0,045307696
914	DB01214	-10,74	71,27	1338,18	0,76	48,19	0,43	Metipranolol	0,046020985
971	DB01299	-9,95	70,67	1312,10	0,67	26,12	0,54	Sulfadoxine	0,04651171
201	DB00353	-11,20	82,89	1514,25	0,99	66,00	0,45	Methylergonovine	0,046923846
1118	DB01567	-10,45	67,72	1279,98	0,94	55,77	0,48	Fludiazepam	0,047053754
215	DB00369	-8,67	68,71	1140,19	1,17	8,58	1,75	Cidofovir	0,04737562
929	DB01233	-9,88	74,50	1236,25	0,85	32,01	0,77	Metoclopramide	0,047665715
740	DB01007	-10,94	71,53	1410,47	0,95	51,90	0,17	Tioconazole	0,047709137
3603	DB04571	-9,63	66,74	1045,00	0,63	65,34	0,17	Trioxsalen	0,047802538
814	DB01096	-9,60	73,75	1220,32	1,01	41,15	1,12	Oxamniquine	0,04852119
146	DB00291	-10,14	70,10	1166,46	0,89	58,52	0,57	Chlorambucil	0,048961222
316	DB00495	-9,09	72,70	1161,85	0,89	14,70	1,27	Zidovudine	0,04904008
1126	DB01582	-9,36	66,93	1179,28	0,68	36,40	0,34	Sulfamethazine	0,04912597
411	DB00612	-10,45	84,08	1405,06	1,07	43,94	0,30	Bisoprolol	0,049145907
153	DB00299	-8,77	63,87	1134,68	0,88	12,28	1,63	Penciclovir	0,04916385
543	DB00771	-10,90	81,24	1569,10	1,16	79,60	0,02	Clidinium	0,04917726
673	DB00927	-9,34	74,02	1247,10	0,99	10,06	1,29	Famotidine	0,049295127
3893	DB06803	-10,29	76,08	1304,49	1,20	47,49	1,35	Niclosamide	0,049693078
780	DB01058	-11,09	76,19	1422,62	1,00	80,56	0,61	Praziquantel	0,0500409
540	DB00768	-11,49	80,28	1515,71	1,00	74,01	0,70	Olopatadine	0,05020711
610	DB00849	-9,60	73,25	1118,38	0,84	48,75	0,37	Methylphenobarbital	0,050239056
333	DB00518	-8,98	66,67	1131,95	0,56	29,40	0,36	Albendazole	0,050257474
655	DB00908	-10,05	78,16	1447,79	0,93	55,09	0,71	Quinidine	0,05030662
737	DB01004	-8,28	71,74	1088,44	1,18	0,00	1,89	Ganciclovir	0,051116765
559	DB00790	-11,68	86,18	1589,38	1,52	57,59	1,78	Perindopril	0,052018374
173	DB00322	-8,28	72,75	1061,47	0,97	7,91	1,29	Floxuridine	0,052619845
364	DB00552	-8,44	72,65	1192,60	1,08	3,35	1,68	Pentostatin	0,052815408
453	DB00664	-9,09	71,88	1158,90	0,66	24,43	0,44	Sulfametopyrazine	0,052829117
154	DB00300	-8,99	77,10	1189,09	1,23	8,58	1,44	Tenofovir	0,053086877
790	DB01068	-10,46	72,60	1365,79	1,12	49,85	1,09	Clonazepam	0,053309977
95	DB00231	-10,70	76,93	1266,15	1,05	70,17	0,82	Temazepam	0,0533475
262	DB00427	-10,69	78,54	1246,44	0,76	78,09	0,00	Triprolidine	0,053792864
137	DB00281	-9,29	67,72	1015,12	0,50	58,27	0,03	Lidocaine	0,05387029
3829	DB04878	-8,72	68,97	1165,45	1,32	4,76	2,04	Voglibose	0,05435267
915	DB01215	-11,08	72,29	1280,91	0,55	67,49	0,00	Estazolam	0,054531813
80	DB00211	-8,98	66,98	1142,07	0,95	18,82	1,45	Midodrine	0,05517006
940	DB01246	-10,77	78,25	1245,20	0,70	61,50	0,00	Trimeprazine	0,05555013
839	DB01131	-8,81	63,05	1054,80	0,48	36,81	0,41	Proguanil	0,055686772
605	DB00842	-10,13	73,44	1242,26	1,33	67,88	0,75	Oxazepam	0,055726975
386	DB00582	-11,27	87,17	1502,04	0,94	50,05	0,20	Voriconazole	0,055819005
603	DB00839	-10,91	77,37	1295,48	0,71	54,60	0,28	Tolazamide	0,056167364
110	DB00249	-8,11	66,44	1034,98	0,86	10,12	1,32	Idoxuridine	0,056531876
754	DB01024	-10,78	77,75	1416,01	1,17	52,42	1,10	Mycophenolic acid	0,057125837
773	DB01048	-10,43	74,67	1301,97	0,87	39,27	0,70	Abacavir	0,057296097
320	DB00499	-10,16	69,59	1181,31	0,93	49,72	1,06	Flutamide	0,057313085
939	DB01245	-9,10	65,58	1088,03	0,51	45,17	0,00	Decamethonium	0,057662606

1125	DB01581	-9,05	65,91	1134,77	1,00	28,05	1,21	Sulfamerazine	0,05823803
238	DB00400	-11,02	77,34	1434,03	0,74	39,10	0,20	Griseofulvin	0,058549643
64	DB00187	-10,55	73,80	1300,82	0,93	48,25	0,52	Esmolol	0,05873984
128	DB00271	-9,39	60,82	1224,73	0,85	32,63	0,83	Diatrizoate	0,05946359
166	DB00315	-10,47	79,00	1277,91	0,90	46,00	0,36	Zolmitriptan	0,05972156
861	DB01154	-9,05	65,03	1056,08	0,52	46,51	0,17	Thiamylal	0,060121328
573	DB00807	-10,50	76,19	1288,96	0,85	40,66	0,74	Proparacaine	0,060261846
495	DB00712	-9,79	70,05	1089,97	0,74	75,43	0,39	Flurbiprofen	0,06030169
642	DB00891	-9,07	70,90	1050,76	0,57	40,61	0,31	Sulfapyridine	0,060728878
244	DB00408	-11,44	79,75	1383,92	0,62	56,47	0,00	Loxapine	0,060879707
298	DB00473	-10,58	71,83	1152,02	0,55	70,76	0,11	Hexylcaine	0,061187804
977	DB01325	-9,37	68,86	1141,67	0,92	31,07	1,23	Quinethazone	0,06139779
77	DB00208	-9,16	64,25	1026,44	0,49	56,63	0,00	Ticlopidine	0,061486483
954	DB01262	-7,80	68,44	1007,58	0,83	4,17	1,23	Decitabine	0,06150651
72	DB00202	-8,74	68,52	1254,83	0,81	8,15	0,39	Succinylcholine	0,06178692
827	DB01114	-9,37	65,02	1150,92	0,88	66,52	0,00	Chlorpheniramine	0,062013686
505	DB00725	-11,04	75,10	1336,64	0,74	52,02	0,46	Homatropine Methylbromi	0,06227541
1135	DB01598	-8,95	75,22	1244,22	0,99	15,66	1,13	Imipenem	0,062295288
683	DB00939	-10,25	73,11	1134,21	0,79	61,71	0,51	Meclofenamic acid	0,062808335
941	DB01247	-8,99	61,70	1047,97	0,60	47,35	0,40	Isocarboxazid	0,0631845
621	DB00863	-9,86	75,50	1299,56	0,89	18,57	0,97	Ranitidine	0,06337488
1034	DB01426	-11,27	76,89	1461,23	0,96	61,54	0,52	Ajmaline	0,06357095
150	DB00296	-10,63	74,19	1185,53	0,67	69,16	0,01	Ropivacaine	0,06376156
326	DB00507	-10,01	68,78	1287,78	1,07	37,88	1,83	Nitazoxanide	0,06380239
336	DB00521	-10,83	75,54	1304,91	0,80	49,58	0,60	Carteolol	0,06393218
639	DB00885	-8,94	66,89	1059,01	0,61	38,49	0,44	Pemirolast	0,06431523
374	DB00564	-10,11	73,71	1058,06	0,56	70,35	0,06	Carbamazepine	0,06447044
557	DB00787	-7,62	68,14	1030,57	1,01	0,00	1,25	Aciclovir	0,06448388
734	DB01001	-9,22	65,88	1052,64	0,84	45,35	0,96	Salbutamol	0,06455535
947	DB01253	-11,56	83,84	1435,40	0,89	65,11	0,55	Ergonovine	0,064644635
866	DB01161	-9,20	70,45	1116,22	0,74	37,45	0,41	Chloroprocaine	0,064646274
1133	DB01595	-9,94	71,85	1324,47	1,38	48,41	1,02	Nitrazepam	0,066090256
579	DB00814	-10,50	74,13	1417,21	0,94	38,06	1,07	Meloxicam	0,0662159
1154	DB01620	-9,73	69,13	1065,53	0,78	69,42	0,00	Pheniramine	0,066239655
74	DB00205	-9,46	70,08	1082,29	0,89	56,51	0,26	Pyrimethamine	0,06665817
784	DB01062	-11,70	85,53	1578,25	0,95	76,18	0,04	Oxybutynin	0,06688303
518	DB00745	-10,38	71,90	1167,12	1,10	74,26	1,13	Modafinil	0,067065775
1031	DB01423	-8,41	63,24	1065,67	0,94	13,87	1,66	Stepronin	0,06748256
216	DB00370	-10,83	75,76	1196,10	0,56	69,56	0,01	Mirtazapine	0,06766501
152	DB00298	-11,86	77,03	1485,04	0,62	56,63	0,00	Dapiprazole	0,067690164
279	DB00446	-10,14	71,55	1255,19	1,48	40,10	2,15	Chloramphenicol	0,06779027
998	DB01359	-11,07	77,89	1269,38	0,69	82,16	0,16	Penbutolol	0,06787479
651	DB00902	-10,87	79,50	1254,06	0,74	62,68	0,00	Methdilazine	0,06830013
934	DB01239	-10,65	79,14	1234,28	0,87	80,47	0,00	Chlorprothixene	0,0685513
3863	DB06701	-9,25	67,79	1008,57	0,53	63,74	0,23	Dexmethylphenidate	0,06867537
257	DB00422	-9,25	67,79	1008,57	0,53	63,74	0,23	Methylphenidate	0,06867537
220	DB00377	-11,14	72,13	1323,28	0,72	83,25	0,16	Palonosetron	0,068783015
5553	DB08799	-10,75	72,74	1199,43	0,64	71,63	0,00	Antazoline	0,06939915
957	DB01265	-8,39	68,39	1038,50	0,80	15,05	1,27	Telbivudine	0,06949189
671	DB00925	-11,01	71,16	1266,45	0,68	71,67	0,00	Phenoxybenzamine	0,07009825
731	DB00998	-9,28	72,21	1108,76	0,84	49,19	0,89	Frovatriptan	0,07100481
1018	DB01407	-9,02	67,26	1042,72	0,63	45,08	0,34	Clenbuterol	0,07106486
3830	DB04880	-9,12	69,28	1076,09	0,83	34,74	1,00	Enoximone	0,071314335
593	DB00829	-10,71	73,12	1187,89	0,66	68,50	0,23	Diazepam	0,071484864
196	DB00348	-11,31	80,74	1422,17	1,64	52,98	2,21	Nitisinone	0,071513355
225	DB00384	-9,17	70,48	1176,51	0,97	40,74	0,88	Triamterene	0,07161993
358	DB00546	-11,83	79,63	1499,73	0,72	73,10	0,00	Adinazolam	0,07231593
995	DB01354	-9,95	78,97	1157,55	1,07	53,77	0,32	Heptabarbital	0,07241717
425	DB00630	-7,96	74,31	1039,24	1,32	0,00	1,82	Alendronate	0,07401624
3869	DB06711	-9,48	63,26	968,34	0,40	73,47	0,00	Naphazoline	0,074202746
300	DB00475	-10,88	76,34	1284,55	0,85	60,35	0,24	Chlordiazepoxide	0,074967235
695	DB00956	-11,11	83,82	1336,66	1,08	68,80	0,65	Hydrocodone	0,076345086
187	DB00338	-11,14	72,81	1483,65	0,62	33,14	0,43	Omeprazole	0,07654762
511	DB00736	-11,14	72,81	1483,65	0,62	33,14	0,43	Esomeprazole	0,07654762
309	DB00487	-10,32	73,85	1450,44	0,81	30,01	0,77	Pefloxacin	0,07689339
355	DB00543	-11,17	79,80	1314,27	0,73	61,96	0,00	Amoxapine	0,07713193
304	DB00480	-9,81	75,13	1145,99	0,99	40,47	1,09	Lenalidomide	0,07713422
575	DB00809	-11,03	78,89	1268,74	0,71	60,11	0,29	Tropicamide	0,07713497
529	DB00756	-10,81	71,75	1287,00	0,94	53,53	0,91	Hexachlorophene	0,077397436

289	DB00461	-9,95	67,71	1040,88	0,66	74,65	0,18	Nabumetone	0,077774286
547	DB00776	-10,29	76,91	1170,38	1,03	56,79	0,67	Oxcarbazepine	0,07782602
1923	DB02546	-10,44	72,60	1184,39	0,95	57,15	0,92	Vorinostat	0,07855189
733	DB01000	-10,70	75,06	1410,69	1,09	37,28	1,70	Cyclacillin	0,07886514
833	DB01123	-9,06	65,32	978,42	0,50	57,32	0,17	Proflavine	0,079057574
585	DB00821	-10,43	73,03	1172,50	0,94	62,38	0,65	Carprofen	0,07937583
274	DB00440	-9,66	64,93	1306,35	0,83	24,26	0,78	Trimethoprim	0,07955256
1149	DB01614	-11,27	74,25	1389,16	0,77	51,54	0,52	Acepromazine	0,07964867
550	DB00779	-8,86	60,63	1074,97	0,96	31,31	1,47	Nalidixic Acid	0,08057326
141	DB00286	-12,70	83,19	1502,10	1,30	86,53	1,36	Conjugated Estrogens	0,080595195
1143	DB01608	-11,76	86,53	1544,12	0,92	59,75	0,15	Propericiazine	0,080673695
1019	DB01408	-10,78	81,40	1590,90	0,90	41,52	0,36	Bambuterol	0,080685854
963	DB01280	-9,40	68,93	1301,76	1,06	0,00	1,92	Nelarabine	0,08075315
678	DB00933	-11,77	77,43	1570,85	0,82	58,96	0,38	Mesoridazine	0,08109039
462	DB00674	-10,95	81,53	1264,23	0,76	54,75	0,41	Galantamine	0,08109531
158	DB00306	-9,09	67,31	1121,11	0,80	38,99	0,38	Talbutal	0,0815922
1020	DB01409	-10,84	84,62	1585,92	0,85	38,57	0,48	Tiotropium	0,0817067
3843	DB05271	-11,11	71,70	1341,38	0,95	65,53	0,26	Rotigotine Transdermal Pa	0,08285329
322	DB00501	-7,68	66,02	1060,55	0,42	6,31	0,00	Cimetidine	0,08312982
1014	DB01399	-10,34	71,98	1188,70	1,29	52,78	1,51	Salsalate	0,08323324
996	DB01355	-9,25	73,11	1046,58	0,81	50,06	0,31	Hexobarbital	0,08333406
389	DB00586	-10,27	75,53	1133,50	0,95	61,75	0,63	Diclofenac	0,08383277
169	DB00318	-11,11	85,83	1321,55	1,14	69,27	0,71	Codeine	0,08417073
245	DB00409	-10,51	66,40	1362,58	0,94	37,60	1,00	Remoxipride	0,08497962
545	DB00774	-9,80	77,52	1273,61	1,10	18,12	1,30	Hydroflumethiazide	0,0852679
1146	DB01611	-11,17	79,55	1402,17	0,92	48,97	0,27	Hydroxychloroquine	0,08534214
206	DB00359	-8,64	69,32	1057,73	0,61	29,35	0,34	Sulfadiazine	0,085657924
763	DB01035	-8,86	67,90	1061,78	0,73	36,70	0,50	Procainamide	0,0860866
946	DB01252	-11,95	82,87	1411,27	1,05	80,83	0,81	Mitiglinide	0,08725187
626	DB00870	-9,08	65,19	1115,87	0,76	58,07	0,42	Suprofen	0,08799219
910	DB01210	-11,16	74,61	1290,98	0,73	64,55	0,34	Levobunolol	0,0881176
268	DB00434	-11,06	79,06	1309,28	1,71	102,12	0,00	Cyproheptadine	0,08967477
276	DB00442	-9,67	69,72	1234,03	1,08	21,61	1,65	Entecavir	0,0901545
1036	DB01428	-9,15	65,70	1052,50	0,81	62,83	0,68	Oxybenzone	0,09030789
599	DB00835	-9,23	65,07	1143,56	0,90	65,94	0,00	Brompheniramine	0,09046522
242	DB00405	-9,23	65,07	1143,56	0,90	65,94	0,00	Dexbrompheniramine	0,09046522
684	DB00940	-11,67	78,09	1521,16	0,98	66,57	0,38	Methantheline	0,09073743
431	DB00636	-8,83	63,32	1013,92	0,64	46,05	0,48	Clofibrate	0,09100363
294	DB00469	-9,83	71,65	1366,13	0,92	15,82	1,37	Tenoxicam	0,09104824
3857	DB06694	-10,35	68,78	1087,18	0,59	79,64	0,00	Xylometazoline	0,091106534
3833	DB04896	-10,27	70,30	1073,75	0,63	77,08	0,11	Milnacipran	0,0912565
812	DB01094	-11,12	74,88	1355,29	0,90	47,41	0,96	Hesperetin	0,092532605
3854	DB06335	-11,56	87,32	1411,73	1,01	68,23	0,51	Saxagliptin	0,09277192
301	DB00476	-10,87	72,59	1252,63	0,91	72,00	0,12	Duloxetine	0,09302679
503	DB00721	-8,89	69,66	1063,75	0,80	36,58	0,55	Procaine	0,093780726
809	DB01090	-8,81	65,97	1046,00	0,50	42,50	0,00	Pentolinium	0,094213486
5560	DB08806	-12,08	88,95	1495,99	1,03	66,79	0,74	Roxatidine acetate	0,0944497
421	DB00625	-11,16	75,79	1270,86	0,71	77,81	0,13	Efavirenz	0,094852775
437	DB00643	-11,49	71,82	1351,71	0,68	66,52	0,35	Mebendazole	0,0951744
388	DB00585	-9,31	72,60	1293,58	1,48	0,00	2,22	Nizatidine	0,09520924
556	DB00786	-10,72	71,18	1399,14	1,66	36,40	2,92	Marimastat	0,09585503
3862	DB06700	-10,64	72,97	1128,34	0,60	74,80	0,19	Desvenlafaxine	0,096007705
876	DB01173	-10,97	73,25	1201,33	0,58	75,89	0,00	Orphenadrine	0,09617111
859	DB01151	-10,65	68,06	1211,17	0,94	72,01	0,42	Desipramine	0,09707123
3873	DB06716	-10,95	71,88	1265,75	0,92	63,13	0,73	Fospropofol	0,098305225
548	DB00777	-11,63	69,23	1474,92	0,92	61,16	0,33	Propiomazine	0,09831199
345	DB00532	-8,92	65,57	1001,53	0,76	48,07	0,64	Mephenytoin	0,09855524
3845	DB06148	-10,62	72,37	1187,14	0,74	90,37	0,00	Mianserin	0,0988372
580	DB00815	-9,94	68,30	1058,86	1,26	65,21	1,40	Sodium lauryl sulfate	0,099544466
460	DB00672	-9,02	72,21	1074,89	0,85	38,10	0,49	Chlorpropamide	0,09958136
478	DB00692	-11,03	77,68	1250,64	0,76	66,00	0,25	Phentolamine	0,09993857
625	DB00869	-9,45	69,33	1194,72	1,17	21,51	1,79	Dorzolamide	0,10026732
700	DB00962	-11,37	84,47	1411,94	0,81	46,20	0,24	Zaleplon	0,100702405
352	DB00540	-9,68	62,26	1218,20	0,97	88,72	0,03	Nortriptyline	0,100982875
267	DB00433	-11,93	84,87	1504,99	0,71	50,06	0,00	Prochlorperazine	0,10311213
321	DB00500	-10,61	70,27	1155,40	0,85	66,74	0,90	Tolmetin	0,103197485
674	DB00928	-8,22	70,50	1061,27	1,22	0,00	2,15	Azacitidine	0,103569716
571	DB00805	-11,48	77,12	1341,87	0,69	65,20	0,19	Minaprine	0,10376716
442	DB00649	-8,17	68,53	1009,80	0,78	19,77	0,84	Stavudine	0,10401651

835	DB01125	-10,66	72,58	1150,79	0,71	75,57	0,16	Anisindione	0,10406667
1043	DB01437	-10,04	82,20	1150,46	1,90	73,59	0,34	Glutethimide	0,104266346
724	DB00989	-8,63	66,06	1099,83	0,52	36,52	0,17	Rivastigmine	0,10448161
163	DB00311	-8,40	70,59	1037,34	0,98	22,08	1,01	Ethoxzolamide	0,10559967
863	DB01156	-9,15	67,73	952,28	0,56	63,65	0,12	Bupropion	0,10607886
795	DB01075	-10,35	67,91	1130,43	0,85	85,91	0,04	Diphenhydramine	0,106883496
139	DB00283	-11,65	83,32	1442,66	0,98	78,38	0,00	Clemastine	0,10787952
909	DB01209	-10,37	73,44	1090,99	0,74	77,04	0,09	Dezocine	0,10822168
481	DB00695	-9,81	73,74	1330,98	1,28	28,33	1,60	Furosemide	0,10854611
311	DB00489	-8,65	67,28	1133,62	0,66	33,58	0,49	Sotalol	0,10895702
210	DB00363	-11,50	81,68	1382,85	0,83	61,35	0,02	Clozapine	0,10954291
400	DB00599	-8,74	62,83	1006,04	0,54	46,68	0,23	Thiopental	0,11090347
340	DB00525	-11,59	70,57	1355,57	0,69	75,98	0,00	Tolnaftate	0,11126369
582	DB00817	-11,44	78,19	1360,84	0,89	51,80	0,98	Rosoxacin	0,111337066
1129	DB01588	-11,50	82,28	1384,39	0,89	79,19	0,14	Prazepam	0,11158419
3585	DB04552	-10,76	74,95	1257,43	1,07	47,48	1,43	Niflumic Acid	0,11165744
484	DB00698	-8,60	71,35	1105,74	1,50	9,10	2,21	Nitrofurantoin	0,11242539
1032	DB01424	-8,66	64,61	1023,38	0,54	40,62	0,18	Aminophenazone	0,11265743
1141	DB01606	-9,38	70,89	1243,07	1,12	5,86	2,01	Tazobactam	0,112787366
881	DB01178	-9,29	67,05	1054,36	1,07	43,33	1,44	Chlormezanone	0,114438266
230	DB00391	-10,63	68,65	1400,28	0,88	30,83	1,06	Sulpiride	0,11470944
936	DB01241	-10,47	71,27	1104,74	0,76	74,37	0,46	Gemfibrozil	0,11531708
680	DB00935	-10,51	74,31	1159,37	0,97	74,25	0,17	Oxymetazoline	0,116435945
405	DB00606	-10,88	75,26	1458,90	1,42	43,39	1,69	Cyclothiazide	0,117026836
562	DB00794	-9,06	68,88	998,99	0,75	55,97	0,22	Primidone	0,11809224
328	DB00509	-11,20	90,47	1431,30	1,49	66,80	0,52	Dextrothyroxine	0,11847913
903	DB01203	-11,24	74,41	1350,59	1,18	49,93	1,49	Nadolol	0,119083166
555	DB00784	-10,31	67,75	1080,56	0,81	76,79	0,53	Mefenamic acid	0,1191982
566	DB00800	-11,00	74,23	1273,78	1,10	60,68	0,99	Fenoldopam	0,120464236
716	DB00980	-10,68	75,66	1154,22	0,79	75,46	0,10	Ramelteon	0,12159547
567	DB00801	-11,92	83,90	1443,71	0,88	78,68	0,29	Halazepam	0,12267399
275	DB00441	-8,85	71,87	1112,64	1,15	12,17	1,87	Gemcitabine	0,12318581
3827	DB04876	-11,61	80,67	1338,15	0,71	64,77	0,29	Vildagliptin	0,12445077
721	DB00986	-11,77	81,64	1372,89	0,64	65,46	0,05	Glycopyrrolate	0,124700636
877	DB01174	-8,77	70,55	1076,26	0,72	43,36	0,52	Phenobarbital	0,12543762
867	DB01163	-10,85	71,96	1359,03	0,99	31,89	1,68	Amdinocillin	0,12607366
3823	DB04844	-11,48	75,18	1389,67	0,87	59,75	0,46	Tetrabenazine	0,12620988
315	DB00494	-10,80	73,66	1361,51	1,11	35,16	1,47	Entacapone	0,12729695
281	DB00451	-11,31	74,25	1483,01	1,11	49,80	0,64	Levothyroxine	0,1278562
826	DB01113	-11,49	80,18	1502,67	0,91	40,97	0,36	Papaverine	0,12970471
722	DB00987	-8,41	70,60	1067,83	1,24	5,55	2,10	Cytarabine	0,12984616
84	DB00215	-11,85	87,62	1435,75	0,83	76,83	0,04	Citalopram	0,13132462
878	DB01175	-11,85	87,62	1435,75	0,83	76,83	0,04	Escitalopram	0,13132462
266	DB00432	-10,08	75,86	1254,32	0,97	23,57	1,54	Trifluridine	0,13142917
3887	DB06751	-11,68	92,84	1745,03	1,17	59,95	0,25	Drotaverine	0,13158509
1114	DB01558	-8,89	64,59	1165,87	0,82	46,03	0,31	Bromazepam	0,13183254
193	DB00344	-10,66	68,91	1201,51	0,92	97,00	0,02	Protriptyline	0,13330796
1134	DB01597	-11,34	89,48	1455,48	1,51	51,09	1,34	Cilastatin	0,13376221
652	DB00903	-9,11	67,06	1180,79	1,05	59,14	0,87	Ethacrynic acid	0,13449883
657	DB00911	-8,07	67,54	1007,69	1,21	11,15	1,86	Tinidazole	0,13615373
366	DB00554	-11,54	73,87	1420,76	1,16	44,07	1,73	Piroxicam	0,13638207
261	DB00426	-10,36	75,37	1405,52	0,96	16,87	1,09	Famciclovir	0,13668123
292	DB00464	-10,66	74,80	1158,01	1,04	68,43	0,86	Sodium Tetradecyl Sulfate	0,13689744
253	DB00418	-9,25	73,45	1124,48	1,14	44,67	0,39	Secobarbital	0,13738072
164	DB00312	-8,83	68,23	1013,44	0,84	45,38	0,50	Pentobarbital	0,13878101
992	DB01351	-8,89	67,72	1026,26	0,89	46,94	0,49	Amobarbital	0,13899156
896	DB01195	-12,60	91,65	1682,77	0,76	53,58	0,27	Flecainide	0,1392923
623	DB00867	-11,26	76,30	1290,33	0,96	65,11	0,71	Ritodrine	0,1401195
692	DB00952	-11,45	84,47	1418,02	0,97	51,89	0,23	Naratriptan	0,1409668
921	DB01221	-9,40	74,67	1071,06	1,24	60,73	0,23	Ketamine	0,14200744
872	DB01168	-8,56	61,05	1010,34	0,55	43,39	0,20	Procarbazine	0,14299878
458	DB00670	-12,02	82,30	1558,29	0,68	39,65	0,40	Pirenzepine	0,14316684
99	DB00235	-8,68	66,50	979,70	0,59	45,66	0,27	Milrinone	0,143453
135	DB00279	-11,40	77,62	1390,66	1,03	50,86	0,94	Liothyronine	0,14464137
339	DB00524	-12,24	80,04	1459,68	0,92	58,54	1,00	Metolazone	0,14656186
127	DB00270	-11,97	84,59	1601,63	0,82	37,50	0,13	Isradipine	0,1494278
753	DB01021	-9,54	73,50	1270,91	1,34	23,67	2,09	Trichlormethiazide	0,14979216
162	DB00310	-11,80	76,36	1337,92	1,01	66,49	1,19	Chlorthalidone	0,15006778
1158	DB01624	-12,37	79,27	1632,92	1,00	74,81	0,12	Zuclopenthixol	0,15072158

231	DB00392	-11,55	77,84	1313,51	0,69	73,80	0,00	Ethopropazine	0,15085024
250	DB00414	-11,69	78,86	1361,60	1,02	59,69	1,08	Acetohexamide	0,15099299
643	DB00892	-11,18	80,49	1362,66	1,07	47,59	0,89	Oxybuprocaine	0,15129268
888	DB01186	-11,53	74,36	1324,37	0,76	76,47	0,02	Pergolide	0,1537163
147	DB00292	-8,56	60,94	1085,76	0,47	45,99	0,13	Etomidate	0,15405446
234	DB00395	-8,69	69,03	1153,50	0,98	32,22	0,82	Carisoprodol	0,15423062
5551	DB08796	-12,34	90,77	1715,07	1,16	54,24	0,12	Pipazethate	0,1568735
1105	DB01544	-11,60	83,56	1355,31	0,84	60,62	0,37	Flunitrazepam	0,1575326
944	DB01250	-11,06	77,36	1356,22	1,18	37,95	1,66	Olsalazine	0,15783307
144	DB00289	-10,67	72,06	1112,28	0,71	83,62	0,06	Atomoxetine	0,1592614
497	DB00714	-11,12	74,83	1198,18	0,80	77,22	0,41	Apomorphine	0,15971875
254	DB00419	-7,90	63,76	992,80	1,17	19,15	1,41	Miglustat	0,1602112
522	DB00749	-11,11	69,54	1295,67	1,09	70,01	0,71	Etodolac	0,16040078
965	DB01288	-11,70	78,19	1357,56	1,16	59,91	1,35	Fenoterol	0,16215259
762	DB01034	-9,31	65,01	1003,50	1,14	51,49	1,66	Cerulenin	0,16408953
332	DB00517	-11,08	72,19	1278,57	1,05	71,76	0,48	Anisotropine Methylbromic	0,16454577
492	DB00708	-12,46	89,33	1643,26	1,05	68,85	0,01	Sufentanil	0,16529366
967	DB01291	-8,28	63,91	1052,65	0,79	28,39	1,24	Pirbuterol	0,16611165
130	DB00273	-10,64	74,46	1384,45	1,16	29,66	1,35	Topiramate	0,16719466
852	DB01144	-9,11	82,70	1145,30	2,04	23,46	1,96	Dichlorphenamide	0,16725576
1015	DB01400	-7,74	63,75	987,30	0,49	17,81	0,28	Neostigmine	0,16787055
635	DB00879	-7,52	65,92	1049,72	1,01	4,19	0,80	Emtricitabine	0,16812193
383	DB00578	-11,46	77,02	1596,11	1,34	40,68	2,02	Carbenicillin	0,16890612
412	DB00613	-12,26	83,75	1524,82	0,78	58,51	0,04	Amodiaquine	0,1693829
3876	DB06725	-10,41	70,87	1439,95	1,10	27,11	1,71	Lornoxicam	0,16983661
491	DB00706	-11,39	95,67	1711,89	1,33	56,29	0,60	Tamsulosin	0,17043266
243	DB00406	-12,52	93,44	1671,30	1,54	74,15	0,01	Gentian Violet	0,17077395
454	DB00665	-11,18	77,47	1392,56	1,25	42,05	1,38	Nilutamide	0,17110208
711	DB00974	-9,17	70,60	1240,84	1,40	3,78	2,61	Edetic Acid	0,17167231
96	DB00232	-9,87	74,95	1230,46	1,38	17,85	2,37	Methyclothiazide	0,17253649
523	DB00750	-8,96	67,63	1005,37	0,90	56,03	0,28	Prilocaine	0,17324415
3841	DB05265	-12,58	81,34	1597,14	1,30	76,94	0,99	Ecabet	0,17483559
313	DB00491	-7,42	70,99	1022,19	1,52	0,00	1,53	Miglitol	0,1749506
83	DB00214	-11,90	80,32	1478,86	0,88	51,70	0,59	Torasemide	0,1759144
775	DB01050	-9,26	59,94	917,38	0,59	76,04	0,39	Ibuprofen	0,17641816
350	DB00537	-11,59	85,57	1459,80	0,89	39,06	0,67	Ciprofloxacin	0,17660004
258	DB00423	-8,85	60,82	1067,79	1,07	26,66	1,95	Methocarbamol	0,1773397
817	DB01101	-10,92	82,16	1553,71	1,50	35,87	2,14	Capecitabine	0,18015882
382	DB00577	-10,30	76,38	1430,32	1,03	11,69	1,13	Valaciclovir	0,18032718
136	DB00280	-12,16	69,92	1521,10	0,97	70,85	0,27	Disopyramide	0,18043137
969	DB01296	-6,94	61,86	899,29	0,99	0,00	1,58	Glucosamine	0,18173555
396	DB00594	-7,03	55,59	952,34	0,65	5,57	0,83	Amiloride	0,18317112
919	DB01219	-11,48	81,70	1425,92	1,06	40,18	1,20	Dantrolene	0,18319064
848	DB01140	-11,49	82,19	1526,32	1,26	34,12	2,09	Cefadroxil	0,18378869
649	DB00899	-12,18	89,96	1612,27	1,11	60,22	0,34	Remifentanil	0,18389297
766	DB01039	-12,14	87,84	1508,67	0,97	76,85	0,12	Fenofibrate	0,18398741
239	DB00402	-11,12	84,43	1643,07	0,60	16,11	0,40	Eszopiclone	0,18628529
413	DB00614	-8,11	67,60	1002,14	1,20	10,66	1,99	Furazolidone	0,18727425
982	DB01333	-12,04	79,31	1453,28	1,18	47,68	1,78	Cefradine	0,18776545
899	DB01198	-11,12	84,56	1642,93	0,60	16,10	0,40	Zopiclone	0,18788117
227	DB00387	-11,53	77,84	1238,00	0,61	82,45	0,00	Procyclidine	0,18849209
319	DB00498	-10,28	70,45	1009,23	0,63	84,43	0,23	Phenindione	0,18874979
917	DB01217	-11,51	80,45	1297,75	0,78	74,36	0,00	Anastrozole	0,18884692
63	DB00186	-11,26	79,84	1253,04	0,94	71,05	0,44	Lorazepam	0,18899393
3849	DB06262	-7,83	65,93	962,28	1,16	14,60	1,68	Droxidopa	0,190463
506	DB00727	-7,54	62,97	987,27	1,64	0,00	2,70	Nitroglycerin	0,19082695
138	DB00282	-7,41	68,72	950,67	1,43	0,00	2,15	Pamidronate	0,19091818
365	DB00553	-8,78	69,15	1013,89	1,20	35,23	1,46	Methoxsalen	0,19129544
470	DB00683	-11,85	79,12	1380,53	0,77	73,22	0,00	Midazolam	0,19208145
433	DB00639	-12,29	82,94	1495,52	0,76	64,60	0,00	Butoconazole	0,193041
968	DB01295	-11,96	79,83	1510,40	0,97	55,58	0,35	Bevantolol	0,19414988
126	DB00269	-12,51	82,78	1630,39	1,04	76,33	0,14	Chlorotrianisene	0,19419476
841	DB01133	-9,08	75,78	1106,26	1,66	22,19	2,21	Tiludronate	0,19486538
714	DB00977	-12,16	83,65	1330,12	0,78	103,58	0,10	Ethinyl Estradiol	0,19534579
879	DB01176	-11,16	76,35	1199,45	0,74	82,17	0,00	Cyclizine	0,19631699
779	DB01057	-7,23	57,79	942,85	0,66	11,09	0,81	Echothiophate	0,19654652
893	DB01192	-11,47	81,00	1351,14	1,16	58,37	0,93	Oxymorphone	0,19691318
5555	DB08801	-11,38	79,18	1290,62	0,93	94,71	0,07	Dimethindene	0,19878003
263	DB00428	-8,64	71,53	1112,24	1,46	0,00	2,71	Streptozocin	0,19936365

54	DB00175	-12,58	94,44	1779,05	2,48	70,34	0,68	Pravastatin	0,19947904
818	DB01102	-12,24	81,88	1417,87	1,05	65,37	1,03	Arbutamine	0,19984439
498	DB00715	-11,96	79,22	1449,69	0,90	64,92	0,24	Paroxetine	0,20041066
175	DB00325	-6,85	57,79	950,74	1,32	0,00	1,71	Nitroprusside	0,20108342
574	DB00808	-12,52	82,59	1458,94	1,07	62,70	1,29	Indapamide	0,2012249
597	DB00833	-12,08	76,62	1466,04	1,31	43,66	2,33	Cefaclor	0,20122927
768	DB01043	-9,42	65,33	916,69	0,60	82,03	0,00	Memantine	0,20181811
586	DB00822	-7,65	59,90	975,07	0,32	21,55	0,00	Disulfiram	0,20384988
1009	DB01392	-12,79	79,98	1563,90	0,84	62,71	0,65	Yohimbine	0,20559126
781	DB01059	-10,84	71,79	1433,51	1,03	23,81	1,58	Norfloxacin	0,20695627
613	DB00853	-7,17	67,99	980,51	1,40	0,00	1,49	Temozolomide	0,2070806
218	DB00372	-12,37	90,89	1599,67	0,86	46,05	0,00	Thiethylperazine	0,2071541
318	DB00497	-11,88	84,47	1378,42	0,98	62,13	0,70	Oxycodone	0,20725754
831	DB01120	-11,73	80,36	1353,45	1,11	65,65	0,89	Gliclazide	0,20812732
483	DB00697	-7,94	64,23	984,18	0,42	26,56	0,00	Tizanidine	0,20905453
469	DB00682	-12,24	83,92	1373,97	0,74	74,62	0,30	Warfarin	0,20929235
57	DB00179	-12,11	77,57	1358,75	1,18	75,62	1,17	Masoprocol	0,21069995
638	DB00884	-8,98	78,29	1106,01	2,07	10,45	3,18	Risedronate	0,21118534
517	DB00744	-8,98	66,99	994,32	0,91	42,04	1,29	Zileuton	0,21259752
513	DB00739	-13,49	81,68	1658,02	1,23	55,76	1,89	Hetacillin	0,21430904
1033	DB01425	-11,36	83,18	1413,40	1,13	36,48	1,34	Alizapride	0,21484339
755	DB01025	-11,44	77,36	1335,29	1,46	65,88	1,24	Amlexanox	0,21514234
1157	DB01623	-12,29	79,89	1826,25	1,35	52,55	0,07	Thiothixene	0,21558666
354	DB00542	-14,36	95,25	1867,23	2,01	79,83	1,83	Benazepril	0,2163733
376	DB00567	-11,69	76,18	1468,88	1,37	38,08	2,09	Cephalexin	0,21658117
252	DB00417	-11,75	75,51	1489,31	1,25	37,12	1,93	Penicillin V	0,21691209
182	DB00333	-12,03	83,90	1389,56	0,83	93,63	0,00	Methadone	0,21835375
488	DB00703	-7,18	63,49	919,17	1,30	5,60	1,84	Methazolamide	0,22271875
906	DB01206	-8,02	62,59	937,21	0,66	31,13	0,72	Lomustine	0,22311005
650	DB00900	-7,73	73,72	1063,53	0,94	10,94	0,82	Didanosine	0,22344685
5557	DB08803	-8,67	62,35	1029,21	0,52	59,48	0,01	Tymazoline	0,22348723
406	DB00607	-13,08	86,49	1762,91	1,55	49,99	2,05	Nafcillin	0,22373176
786	DB01064	-8,21	63,53	954,41	0,94	33,67	1,08	Isoproterenol	0,22567195
3842	DB05266	-8,83	68,88	1014,99	0,67	61,24	0,13	Ibudilast	0,2257024
247	DB00411	-4,32	38,61	631,63	0,73	0,00	1,80	Carbachol	0,22753224
782	DB01060	-11,22	77,97	1553,21	1,09	29,85	1,64	Amoxicillin	0,22795331
554	DB00783	-10,98	77,85	1210,50	1,17	97,17	0,32	Estradiol	0,22894573
369	DB00558	-10,17	73,15	1412,06	1,53	5,89	2,82	Zanamivir	0,22993788
293	DB00467	-9,56	65,80	1415,75	0,95	13,60	1,36	Enoxacin	0,23103958
87	DB00219	-12,29	78,56	1527,93	0,96	65,34	0,23	Oxyphenonium	0,2311587
114	DB00254	-14,22	88,59	1938,38	1,77	52,58	2,63	Doxycycline	0,2317467
390	DB00587	-12,96	97,48	1735,32	1,35	81,51	0,58	Cinalukast	0,23223546
660	DB00914	-8,54	61,53	919,44	0,57	53,54	0,36	Phenformin	0,23239672
576	DB00811	-8,33	76,14	1043,38	1,30	0,00	2,26	Ribavirin	0,23305279
392	DB00589	-12,58	84,11	1520,38	0,74	64,67	0,18	Lisuride	0,23610142
241	DB00404	-11,65	75,72	1306,17	0,79	81,65	0,04	Alprazolam	0,23726183
3837	DB04948	-8,30	67,99	993,76	0,59	35,57	0,00	Lofexidine	0,23765913
3817	DB04837	-11,03	77,31	1210,76	1,04	81,28	0,10	Chlophedianol	0,23813397
647	DB00897	-11,93	79,98	1370,25	0,78	76,24	0,00	Triazolam	0,23844773
303	DB00478	-9,31	66,36	925,77	1,01	80,24	0,36	Rimantadine	0,24358791
493	DB00709	-7,24	65,03	987,00	0,86	5,16	0,64	Lamivudine	0,24414986
224	DB00382	-8,98	66,54	893,48	0,64	66,96	0,33	Tacrine	0,24492264
777	DB01055	-7,06	63,81	905,06	1,16	5,41	1,55	Mimosine	0,24576935
751	DB01019	-4,89	38,84	674,95	0,98	4,44	2,15	Bethanechol	0,24716803
112	DB00252	-10,97	84,30	1271,66	2,14	73,42	1,09	Phenytoin	0,24790302
82	DB00213	-11,50	74,92	1618,04	0,68	24,12	0,37	Pantoprazole	0,2483156
217	DB00371	-8,02	62,63	976,32	1,16	29,21	1,22	Meprobamate	0,24870077
177	DB00327	-11,10	79,18	1308,38	1,45	66,16	0,74	Hydromorphone	0,24870944
514	DB00740	-8,42	74,34	992,12	1,30	30,62	1,16	Riluzole	0,24899247
804	DB01085	-7,97	61,86	947,25	0,73	31,22	0,70	Pilocarpine	0,25030077
463	DB00675	-12,77	94,35	1672,69	1,75	106,64	0,02	Tamoxifen	0,25110078
560	DB00791	-7,65	71,95	995,31	1,14	10,93	0,92	Uracil mustard	0,2528712
532	DB00759	-13,99	90,92	1963,86	2,44	40,79	3,52	Tetracycline	0,2529673
604	DB00841	-11,90	81,18	1354,98	1,16	70,75	0,87	Dobutamine	0,25412604
895	DB01194	-10,62	77,99	1426,36	1,22	17,04	1,56	Brinzolamide	0,2545122
837	DB01127	-12,30	82,56	1465,14	0,81	70,90	0,00	Econazole	0,25538367
894	DB01193	-11,84	78,69	1500,03	1,22	45,14	1,27	Acebutolol	0,25707802
892	DB01191	-9,23	73,44	1017,59	0,93	76,66	0,00	Dexfenfluramine	0,25788862
122	DB00265	-8,86	60,06	919,98	0,55	70,96	0,38	Crotamiton	0,25823492

752	DB01020	-7,33	72,27	947,97	1,55	4,86	1,70	Isosorbide Mononitrate	0,2583354
726	DB00991	-11,98	75,99	1320,40	0,90	83,89	0,48	Oxaprozin	0,2587657
928	DB01231	-12,26	87,74	1349,81	0,88	102,75	0,06	Diphenidol	0,25901037
807	DB01088	-12,63	102,38	1817,60	2,40	100,52	1,22	Iloprost	0,26011017
100	DB00236	-7,59	59,36	1034,68	0,75	19,77	0,90	Pipobroman	0,2609595
570	DB00804	-12,14	81,28	1318,05	0,66	85,12	0,04	Dicyclomine	0,26101053
3890	DB06775	-7,03	67,70	943,51	1,48	1,39	1,62	Carglumic acid	0,26447314
937	DB01242	-11,59	78,87	1297,25	0,87	80,57	0,00	Clomipramine	0,26471275
428	DB00633	-8,97	64,08	933,26	0,83	70,85	0,15	Dexmedetomidine	0,26514566
742	DB01009	-11,32	77,49	1138,64	0,86	86,15	0,66	Ketoprofen	0,2659185
973	DB01320	-13,20	85,39	1507,70	1,09	75,56	1,11	Fosphenytoin	0,2668981
741	DB01008	-7,01	59,44	901,06	0,79	9,76	0,98	Busulfan	0,2693364
1161	DB01628	-12,22	85,13	1421,95	0,82	70,05	0,16	Etoricoxib	0,26997212
994	DB01353	-8,56	72,38	1018,87	1,12	37,11	0,63	Butethal	0,2706781
1128	DB01587	-12,42	72,92	1572,61	0,95	59,84	0,70	Ketazolam	0,27284843
705	DB00968	-8,40	70,09	1003,90	1,49	34,54	1,32	Methyldopa	0,27341747
627	DB00871	-8,24	65,74	1014,91	0,80	39,08	0,78	Terbutaline	0,27453995
538	DB00766	-7,69	73,44	1016,67	1,63	12,09	1,71	Clavulanate	0,27518195
3894	DB06804	-8,98	58,97	1343,30	1,25	23,09	0,33	Nonoxynol-9	0,27585566
399	DB00598	-12,65	84,16	1476,67	1,72	73,29	1,55	Labetalol	0,27850938
3853	DB06288	-11,44	79,48	1529,91	0,99	32,03	0,74	Amisulpride	0,2791527
450	DB00660	-8,53	64,76	991,10	0,78	50,11	0,87	Metaxalone	0,27933502
654	DB00906	-12,50	87,34	1509,75	1,09	57,48	0,87	Tiagabine	0,2816737
577	DB00812	-12,20	74,53	1421,67	0,95	77,61	0,50	Phenylbutazone	0,2824118
185	DB00336	-7,31	63,74	901,71	1,13	11,00	1,63	Nitrofurazone	0,2834242
178	DB00328	-12,45	77,92	1542,22	1,07	60,57	0,85	Indomethacin	0,28444546
104	DB00241	-9,05	76,50	1085,91	1,47	48,85	0,42	Butalbital	0,28674832
821	DB01105	-11,19	73,59	1140,01	0,71	93,80	0,00	Sibutramine	0,2869654
1156	DB01622	-12,67	90,11	1788,10	0,87	36,89	0,08	Thioproperazine	0,2871964
840	DB01132	-12,51	86,56	1520,00	1,23	55,69	1,16	Pioglitazone	0,28928676
284	DB00455	-13,02	90,04	1611,64	0,93	84,91	0,12	Loratadine	0,29039687
828	DB01116	-12,98	84,07	1581,11	0,71	69,17	0,01	Trimethaphan	0,2909698
953	DB01261	-13,04	92,62	1729,47	0,85	51,87	0,17	Sitagliptin	0,29169
306	DB00482	-12,35	79,07	1622,45	1,46	64,89	1,15	Celecoxib	0,2936131
1151	DB01616	-11,76	72,90	1298,03	0,77	89,14	0,00	Alverine	0,29433858
5550	DB08795	-12,28	82,29	1582,93	1,08	36,44	1,40	Azidocillin	0,29579294
1025	DB01414	-10,65	73,48	1443,41	1,29	11,90	2,14	Cefacetrile	0,29780528
427	DB00632	-11,51	90,55	1347,18	1,71	94,20	0,26	Docosanol	0,3002839
961	DB01274	-12,25	83,71	1525,57	1,09	52,90	0,80	Arformoterol	0,30139858
719	DB00983	-12,25	83,71	1525,57	1,09	52,90	0,80	Formoterol	0,30139858
745	DB01012	-12,90	89,18	1587,25	1,11	113,73	0,00	Cinacalcet	0,3022794
331	DB00514	-11,59	77,21	1194,70	0,71	93,20	0,02	Dextromethorphan	0,30666912
887	DB01184	-13,88	97,65	1839,22	1,32	64,10	1,02	Domperidone	0,30817395
794	DB01073	-10,17	77,85	1487,79	1,19	7,64	1,78	Fludarabine	0,30862266
270	DB00436	-13,71	89,73	1667,46	1,24	58,25	1,57	Bendroflumethiazide	0,3087716
1148	DB01613	-9,07	77,09	1218,96	1,96	0,00	3,77	Erythrityl Tetranitrate	0,30888745
527	DB00754	-8,46	68,93	958,70	1,19	43,09	0,94	Ethotoin	0,30911487
179	DB00330	-6,98	54,97	868,58	0,45	16,37	0,60	Ethambutol	0,31000084
665	DB00919	-10,74	76,48	1382,63	1,54	10,80	3,08	Spectinomycin	0,31197047
552	DB00781	-19,73	146,47	3796,71	6,29	32,35	0,41	Polymyxin B Sulfate	0,31235164
307	DB00483	-12,84	88,03	1791,81	1,16	40,88	0,01	Gallamine Triethiodide	0,31459147
583	DB00819	-6,89	62,47	874,23	1,20	6,00	1,63	Acetazolamide	0,31465873
666	DB00920	-11,51	79,73	1307,46	1,14	80,11	0,15	Ketotifen	0,31605613
764	DB01036	-12,26	82,48	1415,56	0,96	101,07	0,01	Tolterodine	0,31723887
1028	DB01418	-13,16	89,35	1584,63	1,15	62,49	0,99	Acenocoumarol	0,32138917
192	DB00343	-10,18	81,55	1594,04	1,97	45,56	0,31	Diltiazem	0,3216363
157	DB00305	-11,13	66,64	1469,74	1,59	17,50	3,24	Mitomycin	0,3219527
55	DB00176	-11,92	85,84	1329,61	0,96	75,61	0,28	Fluvoxamine	0,32200098
634	DB00878	-12,49	107,68	1894,32	2,53	49,93	0,00	Chlorhexidine	0,32349396
563	DB00795	-12,20	88,09	1692,35	1,08	39,62	0,98	Sulfasalazine	0,3237723
987	DB01340	-14,40	86,79	1815,02	1,39	60,22	1,72	Cilazapril	0,32504922
679	DB00934	-11,21	77,80	1231,04	1,16	105,62	0,03	Maprotiline	0,32600015
1160	DB01627	-11,21	76,47	1657,79	0,94	37,28	1,26	Lincomycin	0,32652432
897	DB01196	-13,06	93,18	1729,91	1,25	96,38	0,04	Estramustine	0,3297508
189	DB00340	-11,79	77,99	1315,38	0,89	84,94	0,01	Metixene	0,33218414
5565	DB08811	-12,80	85,86	1649,02	1,12	59,45	0,15	Tofisopam	0,33394125
1132	DB01594	-12,25	81,84	1534,59	1,48	53,27	1,38	Cinolazepam	0,33744758
702	DB00964	-7,70	63,22	976,78	0,70	23,77	0,28	Apraclonidine	0,33834812
237	DB00399	-7,95	76,05	1097,72	1,96	0,00	2,88	Zoledronate	0,3394068

886	DB01183	-12,70	86,52	1428,76	1,07	78,73	0,67	Naloxone	0,33960915
496	DB00713	-13,14	83,58	1687,35	1,18	47,22	1,35	Oxacillin	0,33968383
489	DB00704	-13,16	92,41	1498,17	0,90	75,38	0,45	Naltrexone	0,33969253
750	DB01018	-8,54	69,08	967,84	1,10	47,27	0,61	Guanfacine	0,3411402
709	DB00972	-13,06	92,78	1609,65	0,93	83,00	0,02	Azelastine	0,3412185
297	DB00472	-11,96	81,79	1325,67	0,86	82,18	0,09	Fluoxetine	0,34157938
925	DB01227	-12,76	76,80	1556,19	1,10	82,46	0,05	Levomethadyl Acetate	0,3416283
1039	DB01433	-12,76	76,80	1556,19	1,10	82,46	0,05	Methadyl Acetate	0,3416283
663	DB00917	-13,19	87,09	1522,13	1,04	77,62	0,67	Dinoprostone	0,3433975
3839	DB05246	-8,80	69,69	978,59	1,14	57,96	0,34	Methsuximide	0,34418747
926	DB01228	-12,82	90,37	1550,95	0,92	75,81	0,10	Encainide	0,34612572
718	DB00982	-12,87	78,57	1346,45	1,10	103,72	0,72	Isotretinoin	0,3503664
476	DB00689	-13,28	78,98	1723,73	1,30	47,76	1,69	Cephaloglycin	0,35065567
845	DB01137	-10,93	80,77	1598,06	0,99	23,24	0,82	Levofloxacin	0,35131216
869	DB01165	-10,93	80,77	1598,06	0,99	23,24	0,82	Ofloxacin	0,35131216
207	DB00360	-8,60	70,49	1053,85	1,41	8,06	2,65	Tetrahydrobiopterin	0,3514958
133	DB00276	-13,05	77,63	1705,51	0,98	61,85	0,26	Amsacrine	0,35165018
1139	DB01603	-11,14	76,97	1583,78	1,09	30,73	1,39	Meticillin	0,3520971
1035	DB01427	-7,77	60,34	894,86	0,85	27,73	1,27	Amrinone	0,3528639
853	DB01145	-12,31	92,40	1543,86	1,06	42,65	0,78	Sulfoxone	0,35306963
3836	DB04942	-14,48	87,53	1586,72	1,12	102,18	0,93	Tamibarotene	0,35358208
204	DB00357	-9,16	85,87	1181,15	2,38	63,66	0,54	Aminoglutethimide	0,35527945
844	DB01136	-13,12	88,40	1770,08	1,23	80,58	0,36	Carvedilol	0,35779545
1127	DB01586	-14,42	98,19	1675,43	1,37	112,86	0,55	Ursodeoxycholic acid	0,35893664
820	DB01104	-11,50	76,09	1188,08	0,80	94,96	0,02	Sertraline	0,36359352
397	DB00595	-15,35	94,13	2018,29	2,04	43,39	3,04	Oxytetracycline	0,3638256
3606	DB04574	-13,50	85,92	1464,88	1,16	95,49	0,88	Estropipate	0,3671679
3855	DB06689	-12,86	88,86	1412,41	1,65	82,27	1,55	Ethanolamine Oleate	0,3681562
375	DB00566	-5,68	49,05	737,33	0,75	0,00	1,31	Succimer	0,36843932
338	DB00523	-12,96	79,83	1343,26	1,11	106,08	0,69	Alitretinoin	0,37148607
850	DB01142	-11,61	78,51	1227,39	0,85	90,95	0,02	Doxepin	0,37160963
3851	DB06274	-12,37	93,83	1452,17	2,25	94,29	1,11	Alvimopan	0,37217438
429	DB00634	-7,85	69,85	914,91	1,02	25,89	1,03	Sulfacetamide	0,37231916
553	DB00782	-13,35	87,11	1611,40	0,81	71,06	0,22	Propantheline	0,37300953
1004	DB01380	-13,98	96,34	1775,05	1,35	83,42	0,73	Cortisone acetate	0,37303758
271	DB00437	-5,74	50,14	757,78	0,43	0,64	0,66	Allopurinol	0,37310827
686	DB00943	-7,62	69,23	1008,19	1,18	14,89	0,72	Zalcitabine	0,3783324
854	DB01146	-11,95	80,74	1238,75	0,77	94,95	0,01	Diphenylpyraline	0,37893787
1130	DB01589	-12,68	85,65	1484,01	0,82	76,22	0,00	Quazepam	0,38246936
747	DB01014	-12,91	92,16	1589,40	1,33	50,11	1,37	Balsalazide	0,38595545
194	DB00345	-7,87	66,09	896,48	0,87	30,37	0,97	Aminohippurate	0,38853538
3831	DB04890	-13,50	89,51	1645,79	1,02	66,13	0,62	Bepotastine	0,38867533
630	DB00874	-7,58	55,87	880,67	0,90	24,72	1,60	Guaifenesin	0,39422327
211	DB00365	-11,76	75,60	1572,85	1,01	33,60	1,08	Grepafloxacin	0,39597502
931	DB01235	-7,58	66,25	922,95	1,24	22,22	1,25	Levodopa	0,39708722
59	DB00181	-8,20	64,44	924,57	1,03	43,63	0,81	Baclofen	0,39812487
368	DB00557	-12,91	90,26	1563,87	1,12	75,78	0,40	Hydroxyzine	0,4003253
595	DB00831	-13,34	90,15	1687,70	0,85	61,11	0,00	Trifluoperazine	0,40122485
770	DB01045	-18,66	138,24	3342,79	5,95	66,88	0,44	Rifampin	0,40269056
1006	DB01384	-14,27	96,63	1677,43	0,97	75,39	0,60	Paramethasone	0,40375522
3838	DB04967	-12,43	87,23	1428,56	0,98	75,10	0,27	Lucanthone	0,40420648
334	DB00519	-15,02	92,22	1845,55	1,60	75,43	1,58	Trandolapril	0,40456903
248	DB00412	-12,19	93,35	1508,99	1,23	44,95	0,94	Rosiglitazone	0,4051776
516	DB00742	-6,55	66,67	874,30	1,74	0,00	1,92	Mannitol	0,40638065
123	DB00266	-12,89	85,24	1531,75	1,17	71,96	0,71	Dicumarol	0,4077497
418	DB00620	-14,21	92,24	1700,23	1,13	74,55	0,84	Triamcinolone	0,41061842
923	DB01224	-12,72	88,00	1627,69	1,10	55,44	0,30	Quetiapine	0,41078418
120	DB00262	-6,33	53,98	784,36	0,67	9,49	1,01	Carmustine	0,41090983
858	DB01150	-12,97	84,39	1680,98	1,35	38,79	1,82	Cefprozil	0,4118759
452	DB00662	-12,47	92,72	1678,32	1,23	40,07	0,26	Trimethobenzamide	0,41732848
324	DB00504	-12,66	83,80	1239,33	0,74	110,85	0,04	Levallorphan	0,41895157
310	DB00488	-5,27	46,96	889,29	0,54	0,00	0,66	Altretamine	0,4211977
976	DB01324	-11,83	82,60	1531,18	1,30	25,81	2,02	Polythiazide	0,42221257
291	DB00463	-8,03	70,31	1038,40	1,39	25,20	0,78	Metharbital	0,42260462
66	DB00192	-12,17	81,98	1406,98	1,17	84,41	0,03	Indecainide	0,42376062
344	DB00531	-7,25	66,67	902,88	0,93	15,35	0,79	Cyclophosphamide	0,4237621
3605	DB04573	-12,06	80,60	1259,26	0,91	96,01	0,17	Estriol	0,42591864
537	DB00765	-8,31	65,55	965,63	1,30	44,54	0,90	Metyrosine	0,42890894
5561	DB08807	-13,86	84,33	1686,29	0,73	77,49	0,10	Bopindolol	0,4294578

907	DB01207	-13,05	88,80	1537,80	0,99	73,42	0,38	Ridogrel	0,43001062
816	DB01099	-5,43	44,97	670,86	0,51	5,33	1,05	Flucytosine	0,43272004
908	DB01208	-11,79	82,85	1688,02	0,91	30,69	0,87	Sparfloxacin	0,432928
581	DB00816	-7,67	64,17	955,75	0,79	32,35	0,74	Orciprenaline	0,4345712
343	DB00530	-13,09	86,97	1769,41	1,18	49,40	0,23	Erlotinib	0,43630356
1142	DB01607	-11,63	80,02	1541,75	1,19	20,46	1,91	Ticarcillin	0,43659633
729	DB00996	-7,95	61,67	892,39	0,88	40,45	0,82	Gabapentin	0,43779507
659	DB00913	-13,65	93,94	1579,89	1,02	77,75	0,51	Anileridine	0,4382459
851	DB01143	-6,22	56,30	773,03	0,82	5,94	1,21	Amifostine	0,43987662
662	DB00916	-6,72	59,96	895,49	0,94	7,07	0,88	Metronidazole	0,44094524
515	DB00741	-13,69	90,19	1588,52	1,08	81,25	0,59	Hydrocortisone	0,44362935
644	DB00894	-12,63	83,63	1315,81	0,85	100,27	0,11	Testolactone	0,4450126
494	DB00711	-6,46	54,96	892,93	0,74	11,43	0,84	Diethylcarbamazine	0,44598162
873	DB01170	-7,64	62,73	875,47	0,94	28,72	1,15	Guanethidine	0,44663525
356	DB00544	-5,47	46,05	672,28	0,55	5,37	1,08	Fluorouracil	0,44841167
3848	DB06209	-13,17	89,03	1567,17	0,94	73,11	0,26	Prasugrel	0,45154408
134	DB00277	-6,67	64,14	973,65	1,20	0,00	0,65	Theophylline	0,45208982
78	DB00209	-9,86	85,16	1576,87	2,50	68,08	0,23	Trospium	0,45372966
645	DB00895	-13,47	100,87	1932,58	1,33	60,30	0,83	Benzylpenicilloyl Polylysine	0,45450738
351	DB00539	-13,61	94,30	1722,39	1,92	107,45	0,01	Toremifene	0,45467466
1023	DB01412	-6,64	62,13	950,05	1,23	0,00	0,96	Theobromine	0,45520824
308	DB00485	-14,05	88,43	1812,98	1,23	52,52	1,27	Dicloxacillin	0,45627332
1030	DB01422	-7,20	58,49	904,15	0,87	27,92	1,07	Nitroxoline	0,4566698
424	DB00629	-8,17	63,02	888,38	0,92	48,31	0,73	Guanabenz	0,45737168
188	DB00339	-5,24	47,55	666,59	0,64	0,00	1,10	Pyrazinamide	0,458894
578	DB00813	-13,17	80,72	1546,35	0,85	85,82	0,04	Fentanyl	0,46045005
249	DB00413	-7,58	61,79	899,18	0,56	30,54	0,24	Pramipexole	0,46081793
401	DB00600	-8,47	63,30	911,46	0,55	66,60	0,24	Monobenzone	0,46089575
287	DB00459	-13,48	89,51	1465,23	1,53	97,16	1,06	Acitretin	0,4620203
519	DB00746	-12,15	98,53	1843,43	3,28	38,14	0,95	Deferoxamine	0,46304354
676	DB00931	-15,13	98,18	1967,49	1,93	48,03	2,43	Methacycline	0,464795
455	DB00666	-16,81	146,48	3473,84	8,58	41,04	0,75	Nafarelin	0,46494728
611	DB00850	-12,75	90,74	1626,50	1,09	51,01	0,45	Perphenazine	0,46788055
1011	DB01394	-12,75	83,16	1745,67	1,03	40,37	0,41	Colchicine	0,4691531
118	DB00259	-6,99	58,71	821,34	0,85	17,95	1,28	Sulfanilamide	0,4712717
785	DB01063	-13,50	85,87	1744,35	0,93	56,46	0,43	Acetophenazine	0,47128925
160	DB00308	-12,59	89,85	1573,58	1,26	61,51	0,30	Ibutilide	0,4717918
619	DB00860	-13,71	90,97	1596,06	1,11	80,84	0,58	Prednisolone	0,47266802
584	DB00820	-13,69	89,68	1751,89	0,91	57,02	0,39	Tadalafil	0,4727344
205	DB00358	-13,06	91,53	1608,60	1,09	73,96	0,13	Mefloquine	0,47441864
420	DB00624	-12,62	82,97	1254,03	0,87	110,59	0,09	Testosterone	0,47614366
101	DB00237	-8,51	73,88	1034,67	1,47	41,37	0,55	Butabarbital	0,47738534
445	DB00652	-12,18	80,05	1278,36	0,89	98,44	0,07	Pentazocine	0,47929832
959	DB01268	-13,60	87,34	1742,57	0,95	64,22	0,10	Sunitinib	0,48138756
501	DB00719	-12,07	81,59	1307,16	0,93	91,50	0,00	Azatadine	0,4814049
1010	DB01393	-12,91	89,23	1533,11	1,23	70,53	0,65	Bezafibrate	0,48185185
935	DB01240	-12,99	89,67	1505,40	1,12	76,79	0,48	Epoprostenol	0,48205236
479	DB00693	-13,20	92,01	1476,43	0,95	84,95	0,22	Fluorescein	0,48207188
606	DB00844	-13,82	97,04	1564,83	1,10	81,84	0,60	Nalbuphine	0,48296276
1140	DB01605	-13,33	92,84	1844,11	1,26	50,32	1,00	Pivmecillinam	0,48473296
565	DB00799	-12,93	85,78	1512,29	1,11	78,46	0,45	Tazarotene	0,48539314
233	DB00394	-14,43	93,88	1699,50	1,05	82,35	0,55	Beclomethasone	0,4900205
704	DB00967	-11,96	80,75	1312,76	1,03	90,78	0,00	Desloratadine	0,4900353
387	DB00583	-6,05	55,12	770,81	1,12	5,34	1,48	L-Carnitine	0,49047345
107	DB00245	-9,29	78,46	1362,11	1,48	79,97	0,15	Benztropine	0,49105293
568	DB00802	-13,64	86,75	1813,76	0,97	55,00	0,15	Alfentanil	0,49556094
277	DB00443	-14,36	90,84	1708,30	1,05	81,89	0,56	Betamethasone	0,49584666
930	DB01234	-14,36	90,84	1708,30	1,05	81,89	0,56	Dexamethasone	0,49584666
588	DB00824	-7,28	68,94	998,75	1,41	10,02	0,87	Enprofylline	0,496435
477	DB00690	-13,43	84,19	1620,86	0,92	79,85	0,08	Flurazepam	0,49877435
448	DB00655	-11,49	75,53	1202,68	1,08	98,41	0,10	Estrone	0,49951673
